# Interventions to prevent obesity in Latinx children birth to 6 years globally: a systematic review

**DOI:** 10.1017/S1368980023001283

**Published:** 2023-11

**Authors:** Rachel Bleiweiss-Sande, Kara Skelton, Daniel Zaltz, Montserrat Bacardí-Gascón, Arturo Jiménez-Cruz, Sara E Benjamin-Neelon

**Affiliations:** 1 Johns Hopkins Bloomberg School of Public Health, Department of Health, Behavior and Society, 624 N Broadway, Baltimore, MD 21205, USA; 2 Department of Health Sciences, Towson University, Towson, MD, USA; 3 Universidad Autónoma de Baja California, Department of Medicine and Psychology, Tijuana, Mexico; 4 Department of International Health, Johns Hopkins Bloomberg School of Public Health, Baltimore, MD, USA

**Keywords:** Adiposity, BMI, Children, Early childhood, Hispanic, Latin America, Latino, Preschool

## Abstract

**Objective:**

To conduct a systematic review of obesity prevention interventions in Latinx children ages birth to 6 years published in any language from 2010–2020.

**Design:**

We used PubMed, ERIC, PsycINFO, Scopus, Scientific Electronic Library Online (SciELO) and Google Scholar databases to conduct a search on May 1 2020, January 1 2021 and November 1 2022. We included randomised controlled trials, quasi-experimental studies and non-randomised interventions with a control or comparison group that reported measures of adiposity.

**Setting:**

Interventions taking place in the United States, Latin America or the Caribbean.

**Participants:**

Latinx children ages birth to 6 years.

**Results:**

Of 8601 unique records identified, forty manuscripts about thirty-nine unique studies describing thirty distinct interventions in the United States and nine interventions in Latin America and the Caribbean met our inclusion criteria. Interventions were primarily based in early care and education centres (*n* 13) or combined home settings, for example home and community (*n* 7). Randomised interventions taking place in community or home settings were more likely to report significant reductions in adiposity or weight-related outcomes compared to other settings. Using the Cochrane risk of bias tools for randomised and non-randomised studies, we judged thirty-eight randomised trials and nine non-randomised interventions to have a high or unclear risk of bias.

**Conclusions:**

The results highlight a need for more rigorous designs and more effective intervention strategies in Latinx children at risk for having overweight and obesity. Registered with the PROSPERO database for systematic reviews under registration number CRD42020161339.

The prevalence rates of children with obesity have increased rapidly over the past decade in low- and middle-income countries and in Latin America in particular^(1,2)^. According to estimates from 2008–2013, approximately 20 % of children in Latin America had overweight or obesity^(3)^, and projected trends suggest that these numbers may be higher^(1,4)^. Moreover, children in Latin America and the Caribbean have experienced one of the most rapid increases in age-standardised mean BMI over the past decade, with children in this region now ranking among the highest globally in terms of mean BMI^(1)^. At the same time, Latinx children in high-income countries such as the United States (US) are disproportionately affected by obesity^(5)^. From 2017–2018, Latinx children had the highest rate of obesity (25·8 %) among all racial and ethnic groups^(6)^. Given these health disparities, there is a need to identify culturally appropriate, community-engaged approaches to prevent obesity in Latinx children in the US and Latin America.

The early years (<6 years of age), in particular, represent an important period for obesity prevention^(7,8)^. This period can be defined as the years after birth and before entry into kindergarten, which usually occurs before age 6. There is increasing recognition that the first few years of development lay a foundation for most health-related behaviours, including self-regulatory capacities. Having obesity during early childhood may have important short- and long-term health consequences, including greater likelihood of suffering from psychological comorbidities^(9)^, asthma^(10)^ and a greater risk of musculoskeletal problems and metabolic disorders later in life^(11,12)^. In the US, there is some evidence of an overall levelling off of obesity during early childhood in recent years^(13)^, but this trend has not extended to Latinx populations. The prevalence of having obesity among Latinx children 2–5 years old was four times that of their non-Hispanic white peers from 2011 to 2012^(5)^. More recent estimates show that among Special Supplemental Nutrition Program for Women, Infants and Children (WIC) programme participants, 16·4 % of Latinx children 2–4 years had obesity, representing the highest rate among all racial and ethnic groups with the exception of American Indians and Alaska Natives^(6)^.

Several authors have reviewed the body of literature on obesity prevention interventions in Latinx children. However, most are roughly a decade old and in need of an update^(14–16)^. Given the parallel rise in young children with obesity in the US and Latin America, there is a need to update the literature to identify and highlight successful interventions targeting Latinx children during early childhood^(17)^. Therefore, the objective of this study was to systematically review the efficacy and effect of obesity prevention interventions in Latinx children during the early years, ages birth to 6 years.

## Methods

This review is part of a series of reviews that aim to examine obesity prevention interventions in Latinx children from birth to 18 years of age. Given the wide variation in invention types between early childhood and later childhood and adolescence, we conducted a separate review for young children. The protocol for the larger systematic review and meta-analysis is registered with the PROSPERO database for systematic reviews under registration number CRD42020161339 and has been reported elsewhere^(18)^. We conducted this review according to the guidelines specified by the Preferred Reporting Items for Systematic Reviews and Meta-Analyses statement^(19)^.

### Search strategy and selection criteria

We included studies published between 2010 and 2020 to provide an update to the existing reviews that have been published on this topic^(14–16)^. We included articles in peer-reviewed journals published in English, Spanish or Portuguese. We identified studies reporting the results of interventions aimed at preventing obesity in Latinx children that included children ages birth to 6 years at baseline, thereby excluding interventions targeting mothers during pregnancy. We included studies with a minimum of 50 % of children identified as Latinx, a term used to describe the ethnicity of Mexican, Central American, South American and Caribbean origin individuals and those of Latin American descent living in the US and in other countries^(20)^. For interventions that took place in Latin America and Spanish-speaking countries in the Caribbean, we assumed that all children were Latinx, unless stated otherwise.

We included randomised controlled trials, quasi-experimental studies and non-randomised interventions such as natural experiments. We excluded studies without a control or comparison group. We included interventions that targeted obesogenic behaviours or risk factors for obesity, including diet, physical activity, sedentary behaviour, screen time exposure, stress and sleep – or any combination of these behaviours, as well as interventions targeting obesogenic environmental influences, such as community food access, nutrition programmes and policies, and physical activity environments. We did not require that studies report outcome measures related to obesogenic behaviours or environmental influences, but we excluded studies without interventions targeting a behaviour or environmental influence. We included studies that evaluated and reported outcomes such as change in adiposity, measured by age- and sex-standardised BMI (BMI z-score), BMI (BMI), prevalence of overweight and obesity, percentage body fat, waist or hip circumference, skinfold thickness or other anthropometric measures. If a study reported multiple weight-related outcomes, we attempted to extract all relevant quantitative measures. We excluded studies that reported only weight or height^(21,22)^. We included studies with direct measurement of adiposity by researchers, study staff, or clinicians or health care providers only (*v*. parent report). We excluded studies evaluating interventions to treat, rather than prevent, having obesity during childhood and studies targeting children with a specific known medical condition such as diabetes or CVD.

### Search methods

We searched PubMed, ERIC, PsycINFO, Scopus, Scientific Electronic Library Online (SciELO) and Google Scholar databases from January 1 2010 to January 1 2020, using a search strategy developed *a priori* ^(18)^. We selected databases based on expertise of the authors. Due to considerable overlap between two databases – SciELO and LILACS – we searched SciELO only. We included studies published between 2010 and 2020 to provide an update to the two existing reviews that have been published on this topic. We included articles published in peer-reviewed journals only to ensure that the review comprised high-quality research. Searches were conducted on May 1 2020 and repeated on January 1 2021 and November 1 2022. This search strategy used a combination of medical subject headings and keyword terms informed by search strategies used in related systematic reviews^(14–16)^. We translated the final search strategy for each database into Spanish and Portuguese to capture publications written in languages other than English. However, we did not find any non-English studies that met our inclusion criteria. We performed forward and backward citation searches of included studies and reviewed the reference lists of relevant review articles to identify additional publications. We have provided a sample search strategy in English for PubMed and Scopus in Appendix 1.

### Data extraction and management

We uploaded all search results into Covidence Software (Covidence Systematic Review Software, Veritas Health Innovation, Melbourne, Australia), an online tool developed for systematic review management. Two independent reviewers conducted title, abstract and full-text screening to assess article eligibility. The same two reviewers used a pre-piloted and standardised form for data abstraction. Information abstracted during this phase included publication details; country; study; intervention details (setting, content, format, delivery, control or comparison group); baseline child demographics and characteristics (e.g. geographic location, gender, family income, parental education); recruitment and intervention complement rates; weight-related outcomes and method of ascertainment; statistical methods; results; and limitations. For interventions taking place in the US, we also noted any culturally tailored intervention elements, such as the use of *promotoras* (community health educators) to deliver the intervention. The reviewers resolved differences at the screening and abstraction phase through discussion.

We contacted study authors to request missing data regarding child demographics (*n* 3) and adiposity outcome measures for use in calculating effect sizes (*n* 18). Authors of eleven studies did not respond, five responded that data were unavailable, and two responded with the requested data.

### Quality assessment

Two reviewers independently assessed bias for individual studies using the Cochrane Collaboration’s risk of bias tool (ROB) for randomised^(23)^ or non-randomised studies (ROBINS-I)^(24)^, as appropriate. The ROB tool is used to rate studies according to their level of bias (high, low, or unclear) across seven domains: random sequence generation; treatment allocation concealment; blinding of participants and personnel; blinding of outcome assessment; completeness of outcome data; selective outcome reporting; and other sources of bias^(23)^. The ROBINS-I tool also includes seven domains, including confounding; selection of participants into the study; classification of interventions; deviations from intended interventions; missing data; measurement of outcomes; and selection of the reported result^(24)^. We resolved discrepancies in judgements through discussion. If we deemed a study to have a high or unclear risk of bias for two or more criteria, we assigned it an overall ROB of high or unclear. In the case of multiple criteria deemed to be high or unclear for the same study, we assigned an overall ROB based on whichever rating was more frequently assigned for that study. Otherwise, we assigned the study a low ROB.

We summarised the quality of included studies using the Grading Quality of Evidence and Strength of Recommendations approach^(25)^. Grading Quality of Evidence and Strength of Recommendation rates the quality of studies as high, moderate, low, or very low across four areas, including methodological flaws, consistency of results across studies, generalisability to the target population and effect size^(26)^. In order to include all studies in our quality assessment, we took into account the precision of estimates for studies that did not have enough information to calculate an effect size.

### Evidence synthesis

We conducted a narrative synthesis of included studies by intervention characteristics including design (randomised *v*. quasi-experimental), primary setting where the intervention took place (early care and education centres such as Head Start or another preschool facility; community sites such as churches or community centres; WIC clinics; primary care or hospital settings and the home) and behavioural target, including diet-only interventions, diet and physical activity interventions (targeting physical activity, sedentary time or a combination) and multiple targets including diet, activity, screen time and sleep. We further described studies by population characteristics, including age group (mean age < 2 years or mean age > 2 years), number of children, percentage of females and the percentage of Latinx children in the study sample. We also aggregated studies by region (US or Latin America) and country. For studies taking place in the US, we synthesised studies by cultural elements included in the interventions. Finally, we aggregated the studies by the weight or adiposity-related outcomes reported.

Despite widespread adoption of culturally tailored interventions in the US, there are no published guidelines to develop culturally appropriate dietary, physical activity or other weight-related interventions among minority populations in the US^(27)^. However, several publications have reviewed strategies and approaches to developing interventions for specific subgroups, which include cultural adaption through modifications to evidence-based interventions (cultural tailoring); culturally grounded interventions involving active participation from subcultural group members to create intervention materials, and community-initiated indigenous interventions instigated by a community agent^(28,29)^. Due to the heterogeneity of these approaches, we documented all cultural components reported by study authors, which included offering study materials in multiple languages, having bilingual study staff, incorporating culturally tailored intervention elements (such as using programmes developed specifically for Latinx families), developing interventions based on research with members of the study population, reporting parent or caregiver acculturation, reporting parent or caregiver place of birth and employing *promotoras* (health workers from the Latinx community) to lead intervention activities.

As outlined in our protocol paper, we planned to conduct meta-analyses if more than two studies with comparable exposure and outcome variables were available^(18)^. We examined randomised and non-randomised studies separately due to the major methodological differences in these study designs. We also examined post-intervention and follow-up outcomes separately, if available. We used unadjusted outcome estimates to calculate effect sizes. For continuous outcomes (BMI, BMI percentile and BMI z-score), we calculated adjusted, unstandardised mean differences (Hedge’s g) and for dichotomous outcomes (risk of obesity), and we calculated risk ratios and transformed them using the natural log for use in meta-analyses. We combined effect sizes across outcomes using random effects meta-analyses. We computed the between-study variance component (τ^2^) using the restricted maximum likelihood method, which has been demonstrated to perform well in the case of large τ^2^ estimates^(30)^. We also specified a modified Knapp–Hartung adjustment be applied to the se of the overall effect size; this approach corrects for type-I error probabilities in the case of meta-analyses of a small number of studies^(31)^.

We assessed the homogeneity of effects among studies using forest plots and Higgins *I* ^*2*^ statistics^(32)^. We used funnel plots to assess the risk of publication bias. We conducted all analyses in Stata version 16 (StataCorp; Stata Statistical Software: Release 16; 2019). We produced ROB plots using R software (R Core Team; 2013).

Studies included in this review had considerable variation in results and some inconsistency in the direction of the effect for certain outcomes, including BMI, BMI percentile and BMI *z*-score. In addition, bias was present in some of the individual studies. In this situation, meta-analysis is likely to compound the errors and produce a misleading result^(33)^. Given the high clinical, methodological and statistical heterogeneity of included studies, it would be inappropriate to perform a meta-analysis of included studies^(34)^. Therefore, we do not include meta-analysis results in this study.

## Results

### Study selection

**Fig. 1:**
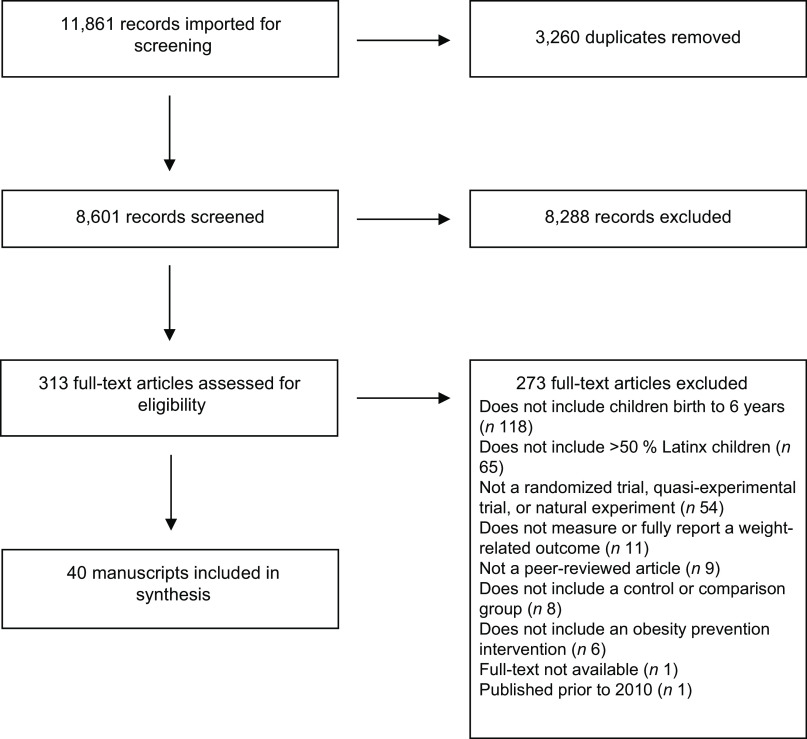
Preferred Reporting Items for Systematic Reviews and Meta-Analysis (PRISMA) flow diagram for the inclusion of studies

Through our literature search, we identified 11 861 records including 3260 duplicates. After deduplication and title and abstract screening, we identified 313 articles that potentially met our eligibility criteria. We have presented full-text exclusions by study in Appendix 2. Of those, forty were included in the systematic review^(35–74)^ and twenty-five were included in meta-analyses (Fig. 1)^(35,36,38,40–42,44–53,55,56,58,59,62,63,67,68,70)^.

### Study design and sample

#### Study design

Table 1 provides an overview of the main characteristics of the included studies. Two manuscripts^(54,72)^ reported findings from the same intervention and study population at different time points, so are treated as a single study in syntheses. Most studies (*n* 30) were randomised or cluster randomised controlled trials^(35–40,43–56,58–62,65,66,68,69)^, and nine were quasi-experimental studies, including one natural experiment^(41)^ and seven non-equivalent group designs^(42,57,63,64,67,70,71,74)^.

**Table 1:** Characteristics of the included studies (*n* 40*)

Article	Study or programme name	City or region, country; intervention setting	Study design	Target population	Behavioural target(s)	Primary outcome(s)	Child characteristics, at baseline			
							Number analysed, intervention, control (unit)	Overall age (mo), mean or range	sd	% of female, overall
Barkin et al. (2012)	Salud Con La Familia (Health with the Family)	NR†, US; community recreation centre	RCT||	Latinx American preschoolers	Child diet, PA, sedentary time	BMI	54, 52 (children)	I**: 50·4	10·8	51
								C††: 49·2	10·8	
Barkin et al. (2018)	Growing Right Onto Wellness (GROW)	Nashville, TN, US; community	RCT	Low-income preschoolers	Child diet, PA, sleep, media use; engaged parenting	BMI	304, 306 (children)	51·6	10·8	52
Bellows et al. (2013)	The Food Friends: Get Movin’ With Mighty Moves	NR, US; urban and rural Head Start centres	RCT	Preschoolers	Gross motor skills, willingness to try new foods	Gross motor skills	132, 131 (children)	NR; I: 53·0	6·8	45
								C: 51·5	6·8	
Berry et al. (2011)	NA‡	North Carolina, US; church and community centre	RCT	Mexican immigrant mothers and their children	Child PA and diet; parent nutrition and exercise knowledge, coping skills, PA	Child BMI %; maternal BMI	28, 28 (children)	37·2	13·2	60
Bonuck et al. (2014)	Feeding Young Children Study (FYCS)	Bronx, NY, US; WIC§ sites	RCT	Parents of infants consuming > 2 bottles of milk or juice/d	Infant bottle use	Bottle use frequency	149, 150 (children)	12·6	0·5	52
Cespedes et al. (2013)	NA	Bogotá, Colombia; preschool facilities	CRCT¶	Preschoolers and their parents and teachers	Child diet and PA	Knowledge, attitudes, and habits	7, 7 (preschools); 622, 594 (children)	36–60		47
Chaparro et al. (2019)	2009 WIC Food Package	Los Angeles County, CA, US; WIC sites	NRS‡‡	Children participating in WIC before and after the WIC package change	Child diet	Weight-for-height z-score, BMIz, obesity at age 4	70 120, 8386, 85 871, 18 241 (children)	0–48		49
Cloutier et al. (2015)	Steps to Growing Up Healthy	Hartford, CT, US; paediatric primary care clinics	NRS	Low-income, racial/ethnic minority preschoolers and their caregivers	Milk consumption (volume and type), juice and SSB consumption, screen time, PA	BMI %	239, 228 (children)	35·4	8·1	41
Cloutier et al. (2018)	Early Childhood Obesity Prevention (ECHO)	Hartford, CT, US; home and community centres	CRTC	Mothers and their newborns attending Brighter Future Family centres	Child SSB intake, introduction of solids, screen time, establishing sleep routines, tummy time as playtime, soothing techniques; maternal diet and PA	Breastfeeding duration; sleep; sleep routines; soothability; screen time; beverage intake	3, 3 (centres) 26, 21 (children)	Newborns		NR
Costa et al. (2017)	NA	São Leopoldo, Brazil; hospital maternity wards	RCT	Low-income mothers and their newborns	Exclusive breastfeeding, limiting added sugars, healthy eating behaviours	Metabolic parameters related to insulin resistance	200, 300 (children)	Newborns		55
Crespo et al. (2012)	Aventuras para Niños	San Diego, CA, US; school, community and home	CRCT	Latinx children	Fam-only: Child diet, PA and TV viewing. Comm-only: Community and school environment and policies	BMIz	3, 3, 3, 4 (schools); 198 (Fam-only), 218 (Comm-only), 165 (Fam + Comm), 227 (children)	70·8	10·8	NR
Davis et al. (2016)	Child Health Initiative for Lifelong Eating and Exercise (CHILE)	NM, US; Head Start centres	CRTC	American Indian and Hispanic children in rural communities	Child diet, PA; Head Start health policies; community food availability and visibility; healthcare provider attention to nutrition and PA	BMIz	8, 8 (centres); 500, 480 (children)	36		47
Fernandez-Jimenez et al. (2019)	The FAMILIA trial	Harlem, NYC, US; public preschools	CRTC	Low-income, underserved preschoolers and parents	Child diet, PA, bodily awareness and emotional regulation	Composite knowledge, attitudes, and habits (KAH) score	9, 6 (preschools); 398, 164 (children)	49·2	7·2	51
Fitzgibbon et al. (2013)	Family-Based Hip-Hop to Health Study	Chicago, IL, US; Head Start centres	CRTC	Hispanic preschoolers and parents	Child diet, PA, television viewing	Feasibility (recruitment and retention)	2, 2 (centres); 72, 74 (children)	54·2	5·0	50
French et al. (2018)	Now Everybody Together for Amazing and Healthful Kids (NET-Works)	St. Paul, MN, US; home and community	RCT	Low-income, racial/ethnic minority preschoolers	Food availability, family meals, television viewing, active play	BMI	265, 269 (children)	40·8	8·4	51
Grummon et al. (2019)	NA	San Mateo County, CA, US; childcare centres	CRTC	Low-income children and parents	Consumption of healthier beverages	Prevalence of OW/OB	2, 2 (centres); 85, 76 (children)	24–60		55
Haines et al. (2013)	Healthy Habits, Healthy Homes	Boston, MA, US; home	RCT	Low-income, racial/ethnic minority children with a TV in bedroom	Family meals, child sleep duration, child’s TV viewing time, elimination of a TV in the room where the child slept	Sleep duration; TV viewing time; presence of TV in child bedroom; family meals	62, 59 (children)	49·2	13·2	52
Haines et al. (2016)	Parents and Tots Together	Boston, MA, US; community health centre	RCT	Racial/ethnic minority families	Child diet, PA, bedtime routines, screen time, identifying hunger and satiety cues; family problem-solving and weight-related behaviours	BMI	56, 56 (children)	43·2	12	48
Heerman et al. (2019)	Competency-Based Approaches to Community Health (COACH)	Nashville, TN, US; home and community	RCT	Racial/ethnic minority preschoolers and their parents	Child diet, PA, sleep, engaged parenting, media use	BMI	59, 58 (children)	50·4	9·6	53
Hughes et al. (2020)	Strategies for Effective Eating Development (SEEDS)	Houston, TX, and Pasco, WA, US; early education centres	RCT	Low-income Hispanic parents and preschoolers	Child self-regulation of energy intake, willingness to try novel foods; parent-child-centred feeding practices	Intervention efficacy	136, 119 (children)	36–60		49
Hughes et al. (2021)	Strategies for Effective Eating Development (SEEDS)	Houston, TX, and Pasco, WA, US; early education centres	RCT	Low-income Hispanic parents and preschoolers	Child self-regulation of energy intake, willingness to try novel foods; parent-child-centred feeding practices	Intervention efficacy	68, 67 (children)	36–60		NR
Linville et al. (2020)	Healthy Balance (HB), Study 2	Pacific Northwest, US; family resource centre	RCT	Rural Latinx immigrant families	Home food environment, PA, sedentary behaviour	Intervention feasibility, efficacy	13, 14 (parent-child dyads)	55·1	14·8	48
Louzada et al. (2012)	NA	São Leopoldo, Brazil, US; home	RCT	Low-income mothers of newborns	Breastfeeding and complementary feeding	Diet, nutritional status, lipid profiles	200, 300 (mother-infant dyads)	Newborns		44
Machuca et al. (2016)	Well Baby Group (WBG)	South Bronx, NY, US; health centre	NRS	Low-income, racial/ethnic minority mothers and infants	Well-childcare + early childhood development, responsive parenting, supportive family relationships, maternal mental health	Rate of overweight and obesity	47, 140 (mother-infant dyads)	6·6	6·8 d	56
Martinez-Andrade et al. (2014),	Creciendo Sanos	Mexico City, Mexico; primary care clinics	CRCT	Parents and preschoolers	Child diet, PA and screen time	Parent report of child’s diet and PA	2, 2 (clinics); 168, 138 (children)	40·6	10·0	46
Natale et al (2014)	Healthy Inside–Healthy Outside (HI-HO)	Miami-Dade County, FL, US; subsidised childcare centres	CRCT	Low-income, racial/ethnic minority preschoolers	Child diet, PA and screen time	BMIz, dietary and PA patterns	6, 2 (centres); 238, 69 (children)	24–60		49
Natale et al. (2017)	Healthy Caregivers–Healthy Children (HC2)	Miami-Dade County, FL, US; subsidised childcare centres	CRCT	Low-income, racial/ethnic minority families with preschoolers	Child diet; parental food preparation and shopping behaviours	Child BMI %; parent report of child dietary habits	28, 16 (centres); 754, 457 (children)	46·72	11·18	50
Palacios et al. (2018)	NA	Hawaii and Puerto Rico, US; WIC sites	RCT	Caregivers of healthy term infants participating in WIC	Breastfeeding, preventing overfeeding, introduction of solid foods, reducing juice consumption	Breastfeeding; introduction of solids; addition of foods to bottle; infant sleep habits; infant weight	102, 200 (caregiver-infant dyad)	1·0	0·45	49
Phelan et al. (2019),	Fit Moms/Mamás Activas	Santa Barbara, San Luis Obispo, and Ventura counties, CA, US; WIC sites	CRCT	Low-income mothers and their infants	Child diet, PA, screen time; changes in home environment	Infant BMIz	5, 6 (clinics); 159, 174 (children)	5·3	3·2	49
Romo et al. (2018)	NA	Cuenca, Ecuador; municipal preschools and home	NRS	Mestizo children	Child diet, PA, screen time	BMIz; weight status; water; SSB and fruit and vegetable consumption; screen time	9, 9 (preschools); 155, 152 (children)	36–48		48
Sadeghi et al. (2019)	Niños Sanos, Familia Sana (Healthy Children, Healthy Family)	Central Valley, CA, US; preschools	NRS	Preschoolers in Mexican-heritage agricultural communities	Child diet, PA	BMIz, log-BMI	387, 313 (children)	71·8	15·7	51
Salazar et al. (2014)	Junta Nacional de Jardines Infantiles (JUNJI)	Santiago, Chile; national daycare centres	CRTC	Preschoolers attending JUNJI day care centres	Child diet, PA	% body fat	2, 2 (day care centres); 120, 145 (children)	52·8	4·8	46
Sangalli et al. 2021	Ten Steps for Healthy Feeding of Children Younger Than Two Years	Porte Alegre, Brazil; healthcare centres	CRTC	Healthcare workers delivering health services to low-income mothers and their infants	Infant feeding practices	Waist circumference, triceps and subscapular skinfolds thickness; energy intake	9, 11 (centres); 373, 363 (mother-infant dyads)	Newborns		NR
Schwartz et al. (2015)	NA	Porto Alegre, Brazil; maternity wards and home	RCT	Adolescent mothers, infants and maternal grandmothers of infants in the same household	Breastfeeding, complementary feeding	Prevalence of overweight and obesity	163, 160 (mother-infant dyads)	Newborns		51
Sharma et al. (2019)	The Texas Childhood Obesity Research Demonstration (TX CORD); Coordinated Approach to Child Health Early Childhood (CATCH EC)	Houston and Austin, TX, US; Head Start centres	NRS	Low-income, ethnically diverse children and parents	Child diet, PA and screen time; preschool environment	Prevalence of obesity	12, 13 (centres); 353, 319 (children)	51·6	8·2	47
Slusser et al. (2012)	Pediatric Overweight Prevention through Parent Training Program (PT)	Los Angeles, CA, US; community sites	RCT	Low-income, Latino families with preschoolers	Caregiver knowledge and skills related to providing healthy diets for their children	BMI	80, 80 (children)	24–48		57
Taveras et al. (2021)	First 1000 d	Chelsea, Revere, Jamaica Plain, and Boston, MA, US; community health centres	NRS	Low-income infants and their mothers	Infant diet, sleep, screen time, developmentally appropriate play; maternal diet, PA, sleep and stress reduction	Infant weight status	1837,1645 (mother-infant dyads)	Newborns		NR
Washio et al. (2017)	NA	Philadelphia, PA, US; WIC sites and home	RCT	Puerto-Rican, breastfeeding mothers	Breastfeeding initiation and continuation	Breastfeeding maintenance	18, 18 (mother-infant dyads)	Newborns		NR
Yin et al. (2012)	Look at Us, We Are Healthy! (Míranos!)	San Antonio, TX, US; Head Start centres	NRS	Mexican American preschoolers	Child diet, PA, screen time, attitudes toward healthy lifestyles	BMIz; weight-for-age z-score; gross motor skills	2, 1, 1 (centres); 179, 80, 83 (children)	4·1	0·6	52
Zaragoza-Cortes et al. (2019)	NA	Yolotepec, Hidalgo, Mexico; community	NRS	Mother-child dyads	Complementary feeding, continued breastfeeding, adequate perception of the child weight, child nutrition	Mother’s perception of child weight status	10, 9 (mother-child dyads)	4·4	1·9	NR

* The review sample included 40 manuscripts reporting findings from 39 unique studies. Hughes et al. 2020 and Hughes et al. 2021 report findings from the same intervention and study population at different time points.

† Not Reported.

‡ Not applicable.

§ Women, Infants and Children Supplemental Feeding Program.

|| Randomised controlled trial.

¶ Cluster randomised controlled trial.

** Control.

†† Intervention.

‡‡ Non-randomised study.

#### Setting

The most common intervention setting was early care and education centres (*n* 13)^(37,40,46–48,50,54,59,60,64,65,67,70)^, followed by community sites (*n* 8)^(35,38,52,55,57,68,71,74)^, WIC clinics (*n* 4)^(39,41,61,62)^, primary care or hospital settings (*n* 4)^(42,44,58,73)^ and the home (*n* 2)^(51,56)^. Eight studies took place in combined settings involving the home and a community, WIC or early care and education setting^(36,43,45,49,53,63,66,69)^.

#### Demographic characteristics of the study population

Studies included 70 458 children at baseline overall, and we included 59 147 intervention children in quantitative analyses. Baseline sample sizes ranged from nineteen^(71)^ to 57 171^(41)^. The mean age of children at baseline ranged from newborns to 70·8 months. Thirteen studies targeted children younger than 24 months of age at baseline^(39,41,43,56,57,61,62,66,69–71,73,74)^. The percentage of females ranged from 47 to 60 %; however, six studies did not report the characteristics of children by gender or sex^(43,45,69,71,73,74)^.

#### Intervention duration and follow-up

Online Supplementary 1 and 2 provide further details of the included studies. Twenty interventions lasted fewer than 12 months in duration^(35,37,38,40,47,48,50–54,58,59,61,63,66,68–70,73,74)^, with the shortest intervention lasting 6 weeks^(58)^. Six interventions lasted 12 months^(39,42–45,62)^, and nine interventions lasted for longer than 12 months^(36,46,49,56,57,60,67,74,75)^. One study reported the results of a natural experiment that followed children for 48 months^(41)^, and three studies did not report the intervention length^(55,65,71)^. Twenty-two studies reported follow-up outcomes ranging from one month to 7 years^(35,36,38,40,41,44,45,47,48,52–60,63,64,66,68,69)^.

#### Behavioural targets

Most interventions targeted a combination of three or more obesogenic factors including diet, physical activity, sedentary time, screen time or media use, parenting skills or the home and community environment (*n* 21)^(35,36,38,42,43,45–49,51–55,58,59,62,63,67,70,74)^; ten interventions targeted diet or infant feeding only^(39,41,44,50,56,61,66,69,71,73)^; four targeted parenting skills and parent feeding behaviour^(54,57,60,68)^, and four targeted diet and physical activity or movement skills only^(37,40,65,75)^. All studies involved parents in one or more intervention elements except one school-based study, which did not engage parents in intervention activities^(63)^.

#### Intervention approach

Of the thirty-nine included studies, six assessed policy, systems or environmental approaches to obesity prevention. The first examined the impact of changes to the 2009 WIC food package through a natural experiment^(41)^. These changes included the addition of fruits, vegetables and whole grains; reduction in the amount of juice, milk, cheese and eggs offered; reductions in the fat levels allowed in milk; inclusion of culturally diverse replacement options; and reduction in the amount of formula for breastfeeding mothers^(41)^. The second study evaluated a beverage intervention targeting changes in children’s food environment, including the adoption and integration of the Healthy Beverages in Childcare Policy in California early care and education centres^(50)^. Two studies by Natale et al. also included changes to policies in early care and education centres. One intervention developed policies to increase physical activity and healthy eating^(59)^, and one integrated the American Academy of Pediatrics Caring for Our Children policies into early care and education centre practices^(60)^. This policy promotes healthy drinks and snacks, adequate physical activity and minimal screen time in childcare centres^(60)^. One study targeted systems-level changes to prevent early childhood obesity through clinical staff obesity prevention training, family-level behavioural knowledge and lifestyle changes and individual-level supports for women and infants considered to be high risk for obesity^(74)^. Finally, a study by Salazar et al. evaluated the impact of a newly developed national preschool education curriculum in Chile^(65)^. Three of these studies found a positive impact on adiposity in favour of the intervention^(60,65,74)^, but two only found an impact among obese children^(60,65)^.

#### Study location

Most studies took place in the US (*n* 30)^(35–39,41–43,45–55,57,59–62,64,67–70,74)^, and nine took place in Latin American countries, including Colombia (*n* 1)^(40)^, Brazil (*n* 4)^(44,56,66,73)^, Mexico (*n* 2)^(58,71)^, Chile (*n* 1)^(65)^ and Ecuador (*n* 1)^(63)^. One study took place in the US and Puerto Rico (included as a US-based study)^(61)^.

#### Cultural components of studies in the US

Of the thirty studies that took place in the US, the percentage of Latinx children ranged from 51^(51)^ to 100 %^(35,38,45,53,54,64,68,69)^. Of these, eight recruited Latinx children exclusively^(35,38,45,53,54,64,68,69)^. Most studies that took place in the US offered study and intervention materials in English and Spanish (*n* 25)^(4,35–39,42,43,45,46,48,49,51–55,59–62,64,67–70)^ and employed bilingual study staff (*n* 21) (Table 2)^(4,35,36,38,39,42,43,45,48,51–55,59–62,64,67,68,70)^. About one-third of these studies (*n* 10) described culturally tailored intervention elements, including programmes developed by the National Latino Children’s Institute^(35)^, provision of Latinx culture-specific foods^(41,55)^ and intervention materials developed for low-literacy populations^(61)^. Eight studies included interventions that were developed or piloted within a similar study population or community^(38,46,48,51,54,55,68,70)^. One study employed *promotoras*, or health workers from the Latinx community, to deliver the intervention^(45)^.

**Table 2:** Cultural components included in studies conducted in the United States (*n* 30)

Article	% Latinx, overall	Study materials offered in multiple languages	Bilingual study staff	Culturally tailored intervention elements	Informed by research with members of the study population	Parent or caregiver acculturation reported	Parent or caregiver place of birth reported	*Promotora*-led intervention activities
Barkin et al. (2012)	100	Yes	Yes	Yes	2	Yes	Yes	2
Barkin et al. (2018)	91	Yes	Yes	2	2	Yes	Yes	2
Bellows et al. (2013)	55 boys, 45 girls	Yes	2	2	2	2	2	2
Berry et al. (2011)	100	Yes	Yes	2	Yes	2	Yes	2
Bonuck et al. (2014)	62	Yes	Yes	2	2	2	2	2
Chaparro et al. (2019)	87	2	2	Yes	2	2	2	2
Cloutier et al. (2015)	82	Yes	Yes	2	2	2	2	2
Cloutier et al. (2018)	60	Yes	Yes	2	2	2	2	2
Crespo et al. (2012)	100	Yes	Yes	Yes	2	2	2	Yes
Davis et al. (2016)	57	Yes	2	Yes	Yes	2	2	2
Fernandez-Jimenez et al. (2019)	54	2	2	2	2	2	2	2
Fitzgibbon et al. (2013)	94	Yes	Yes	Yes	Yes	Yes	2	2
French et al. (2018)	58	Yes	2	2	2	2	2	2
Grummon et al. (2019)	76	2	2	2	2	2	2	2
Haines et al. (2013)	51	Yes	Yes	2	Yes	2	2	2
Haines et al. (2016)	59	Yes	Yes	2	2	2	2	2
Heerman et al. (2019)	100	Yes	Yes	2	2	Yes	2	2
Hughes et al. (2020, 2021)*	100	Yes	Yes	Yes	Yes	2	2	2
Linville et al. (2020)	89	Yes	Yes	Yes	Yes	2	2	2
Machuca et al. (2016)	64	2	2	2	2	2	2	2
Natale et al. (2014)	62	Yes	Yes	2	2	2	Yes	2
Natale et al. (2017)	56	Yes	Yes	2	2	2	Yes	2
Palacios et al. (2018)	60	Yes	Yes	2	2	2	2	2
Phelan et al. (2019)	76	Yes	Yes	2	2	2	2	2
Sadeghi et al. (2019)	100	Yes	Yes	Yes	2	2	2	2
Sharma et al. (2019)	73	Yes	Yes	2	2	2	2	2
Slusser et al. (2012)	100	Yes	Yes	Yes	Yes	Yes	2	2
Taveras et al. (2021)	60							
Washio et al. (2017)	100	Yes	2	2	2	2	2	2
Yin et al. (2012)	90	Yes	Yes	Yes	Yes	2	2	2

* Hughes et al.’s 2020 and 2021 report findings from the same intervention and study population at different time points.

#### Measures of adiposity

The most reported measure of adiposity was BMI z-score (*n* 18), followed by BMI (*n* 13) and BMI percentile (*n* 7). Other adiposity outcomes included risk of overweight and obesity; weight-for-length, weight-for-age and weight-for-height z-score; waist circumference; waist circumference to height ratio; skinfold thickness and body fat percentage. Overall, twelve studies reported a desirable outcome effect in favour of the intervention^(35,38,41,43,51,53,57,63,64,68,74,76)^; two studies reported a positive effect among children having obesity only (in these two studies, all children attending a childcare centre were enrolled in the intervention, regardless of their baseline weight status)^(60,65)^.

### Risk of bias

Online Supplementary 3 and 4 present the overall ROB ratings for the included studies by domain and individual domain ratings by study, respectively. Appendix 3 and 4 provide ROB assessments by study and individual outcome, with reasons. Of randomised studies, eight received an overall high ROB rating^(35,37,46,49,52,56,58,73)^, 20 received an unclear rating^(38–40,43,45,47,48,50,51,53–55,59–62,65,66,68,69)^, and 2 received a low rating^(36,44)^. Among non-randomised studies, one received an overall high ROB rating^(57)^, and eight received an unclear rating^(41,42,63,64,67,70,71,77)^.

### Strength of evidence

Tables 3 and 4 present the strength of evidence by study design and setting for randomised and non-randomised studies, respectfully. Among randomised studies, the strength of evidence was low for all settings, due to risk of bias or indirectness of the evidence. Interventions taking place in community or home settings were the most likely to report a desirable outcome effect. Among the non-randomised studies, the strength of evidence was moderate for early care and education, and insufficient or low for all other settings. Two of the three studies taking place in early care and education settings reported a desirable effect, and one study taking place in a community setting reported a desirable effect. The single studies taking place in primary care, WIC and combined settings all reported a desirable intervention effect.

**Table 3:** Summary of findings for randomised studies, by setting (*n* 30)*

Setting	Participants analysed	Studies	Studies with low/moderate/high risk of bias (*n*)†	% with favourable post-intervention outcome	Strength of the evidence (GRADE)
Early care and education	4195	10	0/8/2	20 %	⊕⊕⊝⊝ Low, due to risk of bias and indirectness of evidence
Combined settings‡	2209	7	1/5/1	14 %	⊕⊕⊝⊝ Low, due to risk of bias and indirectness of evidence
Community	373	5	0/3/2	60 %	⊕⊕⊝⊝ Low, due to risk of bias and indirectness of evidence
Home	465	2	0/1/1	50 %	⊕⊕⊝⊝ Low, due to risk of bias and indirectness of evidence
WIC	601	3	0/3/0	0 %	⊕⊕⊝⊝ Low, due to risk of bias and indirectness of evidence
Primary care	1103	3	1/0/2	33 %	⊕⊕⊝⊝ Low, due to risk of bias and indirectness of evidence

* Hughes et al.’s 2020 and 2021 report findings from the same intervention and study population at different time points so are not counted as independent study populations.

† Assessed using the Cochrane Risk of Bias assessment for randomised trials. Detailed explanation of risk of bias judgements for individual studies is presented in online Supplementary 4.

‡ Combined settings include community and home; community, home and early care and education; primary care and home, and WIC and home.

**Table 4:** Summary of findings for non-randomised studies, by setting (*n* 9)

Setting	Participants analysed	studies	Studies with low/moderate/high risk of bias (*n*)*	% With favourable post-intervention outcome	Strength of the evidence (GRADE)
Early care and education	1704	3	0/3/0	67 %	⊕⊕⊕⊝ Moderate
Primary care	418	1	0/1/0	100 %	⊕⊝⊝⊝ Insufficient
WIC	57 171	1	0/1/0	100 %	⊕⊝⊝⊝ Insufficient
Early care and education and home	276	1	0/1/0	100 %	⊕⊝⊝⊝ Insufficient
Community	1943	3	0/2/1	67 %	⊕⊕⊕⊝ Moderate

* Assessed using the Risk Of Bias In Non-randomised Studies – of Interventions (ROBINS-I) assessment tool. Detailed explanation of risk of bias judgements for individual studies is presented in online Supplementary 5.

## Discussion

In this comprehensive systematic review, we identified forty manuscripts reporting findings from thirty-nine relevant studies reporting adiposity measures from obesity prevention interventions in Latinx children during early childhood. Of the thirty randomised studies included in this review, studies taking place in community or home settings were more likely to report significant reductions in adiposity or weight-related outcomes as a result of the intervention compared to early care and education, WIC, primary care or combined settings. Almost all (*n* 7) of the non-randomised studies reported a significant adiposity or weight-related outcome in favour of the intervention. We found moderate evidence that early care and education settings may be effective in preventing obesity for non-randomised study designs. Overall, we found low or insufficient evidence by setting the effectiveness of obesity prevention interventions in Latinx children, and a lack of consistent exposure and outcome variables prevented further tabulation by study characteristics.

Our findings of low intervention quality and inconsistent results are aligned with previous reviews examining interventions in similar populations. A review of obesity prevention interventions in Hispanic children in the first 1000 d identified only five relevant interventions and found that most were of low or moderate quality^(78)^. Although all but one intervention led to an improvement in the outcome measure assessed, none of the included studies reported change in adiposity or weight-related measures^(78)^. The authors point out that the lack of assessment of any clinical outcome measures was a major limitation of the included studies^(78)^. Branscum and Sharma reviewed obesity prevention interventions in Latinx children from 2000 to 2010^(14)^. Of the nine studies included in their review, two targeted children 6 years and younger. Neither study found an impact on weight-related measures or intermediate outcomes including diet and physical activity^(14)^. A review by Pérez-Morales et al. that focused on obesity prevention interventions in Latinx children in the US from 2001 to 2012 found that the quality of evidence of the included studies was low, with inconsistent improvements in weight-related outcomes^(15)^. Only two studies targeted children 6 years of age and younger, both of which are included in this review^(45,48)^. Two other reviews examining childhood obesity prevention interventions in Latin America and the US focused on school-aged children only^(16,79)^.

Although including interventions that took place in both the US and Latin America in this review represents a strength of our research, this also led to substantial heterogeneity in terms of the child, study and intervention characteristics. Indeed, the major drivers of obesity among Latinx children in the US and Latin America are diverse and may include contributors such as dietary factors, the local food environment and physical activity patterns^(80)^. There is substantial evidence that points to an ongoing shift in dietary intake and energy expenditure in less developed regions such as Latin America, referred to as the nutrition transition^(81)^. Researchers have pointed to broad changes in the food system at the national and local level, which have led to increases in low-nutrient-dense, highly processed food and sugar-sweetened beverage consumption and an uptick in away-from-home eating^(80)^. These dietary shifts are exacerbated by changes to the local food environment including increased access to supermarkets and fast-food restaurants^(80,82)^ and exposure to targeted food and beverage marketing that promotes unhealthful products^(83,84)^. Research has also demonstrated that Latinx children in the US and Latin America may be at risk for physical inactivity due to limited access to greenspace, high neighbourhood crime rates and transportation barriers^(85–87)^.

Factors associated with acculturation may impact weight status among Latinx children in the US, beginning as early as infancy. For example, Latinx mothers are more likely to initiate breastfeeding than the national average, but they are also more likely to supplement with formula feeding, often due to beliefs regarding cultural norms and the need to return to work^(88,89)^. In addition, although recent Latinx immigrants experienced lower rates of chronic disease compared to their non-Latinx white peers, studies have demonstrated that more time spent in the US was associated with having obesity^(90)^. Clearly, the multifaceted nature of factors that may influence obesity in Latinx children necessitates a multidimensional response to obesity prevention.

We acknowledge several limitations to this review. Our decision to focus on studies reporting measures of adiposity as an outcome may lead us to exclude studies focused on strategies to improve behaviours associated with obesity, such as changes in diet, physical activity or sleep. However, other reviews have examined specific obesogenic behaviours, such as sugar-sweetened beverage intake^(91)^ and physical activity^(92,93)^. Second, the limited number of interventions for obesity prevention with available data to compute effect sizes restricted our ability to conduct meta-analyses and the number of reviews with comparable study designs and outcome variables. However, by including all available studies in a narrative synthesis, we have reviewed the available literature as rigorously as possible. Finally, our decision to include both randomised and non-randomised study designs introduced analytic complexities, further precluding meta-analysis. However, by including both randomised and non-randomised study designs, this review sheds light on potential policy, systems and environmental approaches to obesity prevention in Latinx populations.

### Implications for policy and practice

Our review found that interventions taking place in community or home settings were more likely to report significant reductions in adiposity or weight-related outcomes as a result of the intervention compared to early care and education, WIC, primary care or combined settings. Community-based interventions, in particular, involve multiple stakeholders and buy-in from diverse community groups. These interventions may have greater success due more rigorous formative research with the study population and a better understanding of important culturally relevant intervention components. There is a need for more culturally appropriate, community-engaged approaches in future research to address the broad inequities in health in Latinx children.

Studies employing quasi-experimental designs may hold promise for future obesity prevention interventions. Specifically, interventions that use policy, systems and environmental strategies for obesity prevention have emerged as effective strategies to address complex public health issues. These strategies target the broader social and environmental context to support diet and physical activity changes, thus addressing underlying determinants of health and social inequity. Policy, systems and environmental strategies are particularly important for populations at a greater risk of obesity, including Latinx children. Five studies in our review assessed policy, systems or environmental strategies for obesity prevention in Latinx children, with two demonstrating a positive intervention effect among obese children. It is important to note that the effects of obesity prevention interventions may take much longer to appear than treatment interventions. In addition, studies targeting changes in policy or environmental factors may not have noticeable effects in the short term. Longer study duration and long-term follow-up with participants may be necessary to ascertain the true impact of preventative intervention strategies and is critical to advancing successful approaches on a broader scale.

Recent research has highlighted the need to use novel approaches to adapt and scale up intervention strategies for obesity prevention and control in the US and Latin America. Using a case study approach to understand how successful obesity policies and programmes have been implemented in the US and Latin America, Perez-Escamilla et al. found that evidence-based advocacy and evidence of scalability and advocacy were key factors to the launch and implementation of successful interventions^(94)^. The authors argue that the use of implementation science, which aims to promote integration of research findings into policy and practice, may be an important strategy to use during intervention implementation as well as during the maintenance phase to ensure ongoing success and sustainability. Implementation science can offer a forward-thinking approach to designing, implementing and adapting obesity prevention research in Latinx communities.

### Conclusion

In this systematic review and meta-analysis, we found that randomised interventions taking place in community or home settings were more likely to report significant reductions in adiposity or weight-related outcomes compared to other settings. Studies with less-rigorous study designs, such as quasi-experimental studies, were also more likely to report a favourable intervention effect. This review provides an important update to the literature regarding interventions to prevent obesity in Latinx child populations globally over the past decade. Preventing obesity among Latinx children is an issue of critical global public health importance. Results are relevant to stakeholders across multiple sectors engaged in obesity prevention in Latinx children, including community health workers, researchers and policymakers.

## Supplementary material

For supplementary material accompanying this paper visit https://doi.org/10.1017/S1368980023001283. click here to view supplementary material
