# Supplementary material for: Interventions to prevent obesity in Latinx children birth to 6 years globally: a systematic review
Source: Public Health Nutr. 2023 Aug 25;26(11):2498–513. doi: 10.1017/S1368980023001283 (PMC10641617; doi:10.1017/S1368980023001283)
Supplement: Supplementary file 1 [file S1368980023001283sup001.docx]

**SUPPLEMENTARY MATERIAL**

| **Supplement 1.** Summary of study outcomes: number analyzed and intervention duration, follow-up, intensity, outcomes and effect (N = 40)^*^ | | | | | |  |
| --- | --- | --- | --- | --- | --- | --- |
| Article | Total number analyzed (intervention, control) | Duration | Post-intervention follow-up | Dose and intensity | Weight-related outcome, effect | Desirable intervention effect |
| Barkin et al. (2012) | 75 (35, 40) | 4 mo | 3 mo | 12, 90 min sessions | BMI (adjusted): B = –0.59 [95% CI: –0.94 to –0.25]; P = .001 | Yes |
| Barkin et al. (2018) | 610 (304, 306) | 36 mo | 36 mo | 90 min coaching calls 1/wk for 12 wk; 1/mo for 9 mo; 24 mo frequent cues to action | BMI (36 mo, adjusted): B = 0.05 [95% CI, −0.29 to 0.38]; P = .79 BMI trajectory (3-yr): no effect (P = .39) Risk of OB (3-mo post-intervention follow-up, adjusted): RR = 0.51 [95% CI, 0.29- 0.92]; P = .02; corrected P = .10 | No |
| Bellows et al. (2013) | 201 (98, 103) | 4.5 mo | None | 72, 15-20 min lessons | BMI: no effect (regression results NR) BMIz: no effect (regression results NR) | No |
| Berry et al. (2011) | 56 (28, 28) | 6 mo | 3 mo | 1/wk for 3 months, 1/mo | BMI %: Intervention (baseline, follow-up): 86 ± 7.5, 82 ± 6.8 Control: 86 ± 11.1, 88 ± 8.5; P for difference = .03 | Yes |
| Bonuck et al. (2014) | 135 (63, 72) | 12 mo | None | 1/quarter, NR | Weight-for-length > 85th %: OR = 1.02 [95% CI = 0.5-2.0]; P = .95 | No |
| Cespedes et al. (2013) | 858 (433, 425) | 5 mo | 12 mo | Classroom activities: 1 hr/day; 1x 1 hr family workshop; Parents: 3 workshops + weekly health notes; Teachers: 3 training sessions + personalized sessions 2 hr every 2 wk | BMI (6-mo): Intervention (baseline, follow-up): 15.99 ± 0.09, 16.57 ± 0.45; Control: 15.77 ± 0.08, 16.40 ± 0.49; P for difference = .19 BMI (18-mo): no effect (P = 0.5) | No |
| Chaparro et al. (2019) | 182,618 (70120, 8386, 85871, 18241) | 48 mo | 48-60 mo | Ongoing WIC enrollment | Weight-for-height z-score and BMIz: no effect (overall results NR) Risk of OB (at age 4):  Boys: RR = 0.88 [95% CI, 0.86 - 0.91]; P < .05 Girls: RR = 0.90 [95% CI, 0.87 - 0.93]; P < .05 (Outcomes for full, new compared to full, old WIC package) | Yes |
| Cloutier et al. (2015) | 418 (200, 218) | 12 mo | None | 3-5 min session/visit + follow-up phone call | BMI %: B = −0.23 [95% CI, −0.33, −0.13] | Yes |
| Cloutier et al. (2018) | 34 (22, 12) | 12 mo | None | Standard curriculum (1x/wk, 60 min home visits) + 8 enhanced education modules | Weight-for-length BMI (intervention, control):   6-mo: 17.5 ± 1.5, 16.9 ± 2.0, P for difference = .37  12-mo: 17.4 ± 1.6, 17.3 ± 1.0, P for difference = .83  Weight-for-length z-score (intervention, control):   6-mo: 0.4 ± 1.1, 0.9 ± 2.7, P for difference = .61  12-mo: 0.2 ± 1.2, 0.8 ± 2.4, P for difference = .51 | No |
| Costa et al. (2017) | 1 yr: 396 (163, 233) 4 yr: 345 (144, 201) 8 yr: 305 (126, 179) | 12 mo | 7 yr | 40-60 min, 1/mo | BMIz, difference (intervention - control):  Girls, birth to 1 year: 0.11 [95% CI, −0.31, 0.53]; P = .61  Girls, 1 to 4 years: − 0.01 [95% CI, − 0.32, 0.30]; P = .94  Girls, 4 to 8 year: 0.15 [95% CI, −0.14, 0.45]; P = .32  Boys, birth to 1 year: −0.07 [95% CI, −0.40, 0.27]; P = .69  Boys, 1 to 4 years: −0.07 [95% CI, −0.41, 0.27]; P = .67  Boys, 4 to 8 year: −0.29 [95% CI, −0.65, 0.06]; P = .10 | No |
| Crespo et al. (2012) | 441 (96, 83, 128, 134) | 12 mo | 2 yr | Home visits: NR; environmental changes: ongoing; promotora training: 22 hr; teacher training: 5-5.5 hr | BMIz: Comm main effect: B = −0.19, SE = 0.33; P = .54; Fam main effect: .003, SE = 0.33; P = .92 BMI %: Comm main effect: B = 0.20, SE = 0.84; P = .72; Fam main effect: B = .72, SE = 0.85; P = .40 Weight status: Comm main effect: B = −0.19, SE = 0.29; P = .53; Fam main effect: B = .036, SE = 0.29; P = .90 | No |
| Davis et al. (2016) | 1,816 (945, 871) | 24 mo | None | Classroom: 30min/day; staff training: 1/quarter; family and environmental change: ongoing | BMIz (change per 6-month study time): intervention slope = 0.039 [95% CI, 0.014 to 0.063]; comparison slope = 0.038 [95% CI, 0.014, 0.063; difference = 0.011 [95% CI −0.024, 0.046]; P = 0.54 | No |
| Fernandez-Jimenez et al. (2019) | 448 (304, 144) | 4 mo | 1 mo | Children: 37 hr/4 mo; parents: 12 hr/4 mo | BMIz: B = −0.03 [95% CI, −0.29, 0.24]; P = .84 | No |
| Fitzgibbon et al. (2013) | 128 (61, 67) | 14 wk | 1 yr | Children: 40 min, 3x/wk; parent: 90 min, 1/wk | Adjusted difference (intervention - control), post-intervention: BMI: B = −0.04 [95% CI, −0.13, 0.05]; P value NR BMIz: B = −0.03 [95% CI, −0.13, 0.06]; P value NR 1-year follow-up: BMI: B = 0.22 [95% CI, 0.02, 0.41]; P < 0.05 BMIz: B = −0.03 [95% CI, −0.03, 0.17]; P value NR | No |
| French et al. (2018) | 534 (265, 269) | 36 mo | None | Home visits: 1/mo; telephone check-in calls ongoing; parenting classes 12 wk (length NR) | Adjusted difference (intervention - control): BMI: B = –0.19 [95% CI, –0.64, 0.26] BMIz: B = –0.07 [95% CI, –0.23, 0.08] BMI %: B = –0.33 [95% CI, –3.93, 3.26] Waist circumference (cm): B = –0.58 [95% CI, –1.91, 0.76] Triceps skinfold (cm): B = –0.35 [95% CI, –1.62, 0.92] | No |
| Grummon et al. (2019) | 154 (80, 74) | 12 wk | None | Environmental + policy changes: ongoing; educational activities: NR | Adjusted difference (intervention - control): Prevalence of OW/OB: Mean = −6% [95% CI, −15%, 3%] BMI %: 1.2 [95% CI, −2.5, 4.9] BMI: 0.03 [95% CI, −0.2, 0.3] | No |
| Haines et al. (2013) | 111 (55, 56) | 6 mo | None | 4 home visits; 4 health calls; 2x/wk texts for 16 wk; 1/wk text for 8 wk | Adjusted difference (intervention - control): BMI: B = −0.40 [95% CI, −0.79, 0.00]; P = .05 BMIz: B = −0.17 [95% CI, −0.40, 0.07]; P = .17 | Yes |
| Haines et al. (2016) | 9 wk: 94 (46, 48) 9 mo: 96 (46, 50) | 9 wk | 9 mo | 9x, 2 hr sessions | Adjusted difference (intervention - control) Post-intervention: BMI: B = −0.36 [95% CI, −1.23, 0.51); P = 0.41 BMIz: B = −0.25 [95% CI, −0.75, 0.25); P = 0.33 9-mo follow-up: BMI: B = 0.18 [95% CI, −0.58, 0.93); P = 0.64 BMIz: B = 0.06 [95% CI, −0.36, 0.48); P = 0.64 | No |
| Heerman et al. (2019) | 117 (59, 58) | 15 wk (in-person) + 3 mo (phone) | 6 mo | In-person: 1/wk, 90 min; phone-calls: 2/mo, time NR | BMI (adjusted): B = –0.41 [95% CI: –0.82 to 0.01]; P = .05 | Yes |
| Hughes et al. (2020) | 178 (101, 77) | 7 wk | 12 mo | 1/wk | BMIz: no effect (P = 0.8) | No |
| Hughes et al. (2021) | 135 (68, 67) | 7 wk | 12 mo | 1/wk | BMIz: no effect (P = 0.91) | No |
| Linville et al. (2020) | 27 (13, 14) | NR | 4 mo | 6 sessions, 12 hr total | BMI: B = 0.035; SE - 0.114; P = .758 |  |
| Louzada et al. (2012) | 12-16 mo: 396 (166, 230) 3-4 yr: 344 (149, 195) 7-8 yr: 308 (131, 177) | 12-16 months | 3-4 yr and 7-8 yr | 40 min home visits, 1/month for 6 mo and 1x/2 mo for 6 mo | Risk of OW, boys:  12-16 mo: RR = 0.95 [95% CI: 0.65, 1.39] 3 to 4 yr: RR = 1.05 [95% CI: 0.58, 1.89] 7 to 8 yr: RR = 1.20 [95% CI: 0.75, 1.91] Risk of OW, girls:  12-16 mo: RR = 0.99 [95% CI: 0.68, 1.43] 3 to 4 yr: RR = 1.23 [95% CI: 0.68, 2.19] 7 to 8 yr: RR = 1.19 [95% CI: 0.67, 2.10] Risk of OB, boys: 12-16 mo: RR = 0.84 [95% CI: 0.31, 2.22] 3 to 4 yr: RR = 1.31 [95% CI: 0.33, 5.09] 7 to 8 yr: RR = 1.73 [95% CI: 0.77, 3.90] Risk of OB, girls:  12-16 mo: RR = 1.24 [95% CI: 0.55, 2.77] 3 to 4 yr: RR = 3.04 [95% CI: 0.81, 11.31] 7 to 8 yr: RR = 1.24 [95% CI: 0.47, 3.22] | No |
| Machuca et al. (2016) | 187 (47, 140) | 18 mo | 6-12 mo | 11 sessions during well-baby visits | Prevalence of OW/OB: OR = 0.12 [95% CI: 0.02, 0.94] | Yes |
| Martinez-Andrade et al. (2014) | 3 mo: 101 (99, 102) 6 mo: 108 (109, 99) | 6 wk | 3 and 6 mo | NR | BMI, adjusted (3 mo): B = 0.23 [95% CI: –0.07, 0.54] BMI, adjusted (6 mo): B = 0.06 [95% CI: –0.17, 0.23] | No |
| Natale et al. (2014) | 6 mo: 239 (NR) 12 mo: 185 (NR) | 6 mo | 12 mo | Teachers: 2x trainings, time NR; Parents: dinners 1/mo, newsletter 1/mo, at-home activities NR; centers: ongoing environmental changes | BMIz (6 mo): intervention: B = 0.57 [95% CI: 0.24, 0.91]; control: B = 0.25 [95% CI: −0.41, 0.91] BMIz (12 mo): B = 0.72 [95% CI: 0.42, 1.03); B = 0.76 [95% CI: 0.16, 1.35]; P for difference = 0.81 | No |
| Natale et al. (2017) | 1211 (754, 457) | 2 academic yr | T2 (end of 2010-2011 school year), T3 (beginning of 2011-2012 school year), and T4 (end of 2011-2012 school year) | Parents + teachers: 6, 1/mo sessions; child curriculum: weekly (time NR) | BMI % (overall): B = –1.95, SE = 0.97; P = 0.04 BMI %, children with OB: B = –0.23, SE = 0.16; P = 0.003 BMI %, children without OB: B = –0.14, SE = 0.09; P = 0.78 | Yes, OB only |
| Palacios et al. (2018) | 170 (84, 86) | 4 mo | None | Texts 1/wk | Mean (SD) Weight-for-age z-score: intervention: −0.05 (0.85); control: −0.05 (0.85); P for difference = 0.976 Length-for-age z-score: intervention: 0.59 (1.57); control: 0.61 (1.56); P for difference = 0.931 BMIz: intervention: −0.53 (1.38); control: −0.54 (1.39); P for difference = 0.958 | No |
| Phelan et al. (2019) | 332 (173, 159) | 12 mo | None | 4 text messages/wk; group session 1/mo (time NR); ongoing internet content; WIC care | BMIz: B = 0.19 [95% CI: −0.08, 0.46]; P = 0.16 Triceps skinfold for age z-score: B = −0.04 [95% CI: −0.42, 0.35]; P = 0.85 Subscapular skinfold for age z-score: B = −0.30 [95% CI: −0.71, 0.11]; P = 0.16 | No |
| Romo et al. (2018) | 176 (144, 132) | 3 mo | 1 mo | NR | BMIz: B = −0.28 [95% CI: −0.17, −0.40; P < 0.001 | Yes |
| Sadeghi et al. (2019) | 700 (387, 313) | 3 yr | 1 yr | Parents: 1 hr, 1/mo Children: PE classes 20-30 min, 1/wk | BMIz:  Girls: B = 0.30; SE = 0.12; P = 0.01 Boys: B = 0.14; SE = 0.15; P = 0.36 log-BMI: Girls: B = 2.70; SE = 0.02; P < 0.001 Boys: B = 2.69; SE = 0.02; P < 0.001 Waist circumference to height ratio:  Girls: B = 0.48; SE = 0.01; P < 0.001 Boys: B = 0.47; SE = 0.01; P < 0.001 | Yes |
| Salazar et al. |  | NR | None | Parents: Classes 1-2x/month;  Children: Preschool curriculum changes NR | Body fat %:  OB: intervention, -1.5 ± 1.8; control, 1.3 ± 2.0; P < 0.01  Normal weight: intervention, -0.7 ± 2.2; control, 1.0 ± 2.1; P < 0.01  Weight for height z-score:  OB: intervention, -0.1 ± 0.5; control, -0.1 ± 0.7; P = .64  Normal weight: intervention, -0.02 ± 0.4; control, -0.05 ± 0.4; P = 0.56 | Yes |
| Sangalli et al. (2021) | 715 (360, 355) | 1 mo | 6 mo | 1 training session delivered to medical staff | 6 mo outcomes not reported (no difference) | No |
| Schwartz et al. (2015) | 207 (98, 109) | 4 mo | 4-7 yr | 6 counseling sessions (time NR) | Prevalence of OW/OB: RR = 1.24 [95% CI: 0.86, 1.81]; P = 0.254 | No |
| Sharma et al. (2019) | 848 (365, 483) | 2 yr | None | Teacher training: 4-6 hr; classroom environment changes: ongoing | BMIz: B = −0.26 [95% CI: −0.50, −0.01]; P = 0.041 BMI %: B = −6.5 [95% CI: −12.4, −0.69]; P = 0.028 | Yes |
| Slusser et al. (2012) | 121 (61, 60) | 15-17 wk | 12 mo | 9, 90 min classes | BMIz (mean difference between intervention and control): −0.24, SE = 0.11; P = 0.04 | Yes |
| Taveras et al. (2021) | 1837 (1096, 741) | 3 yr | 6 mo, 12 mo | Education materials and 2x/wk texts messages; health coaching phone calls | Weight-for-length z-score: B = −0.27 [95% CI: –0.39, –0.15] | Yes |
| Washio et al. (2017) | 36 (18, 18) | 6 mo | 6 mo | Financial incentives 1/mo | Weight-for-age z-score: B = −0.14 [95% CI: –0.81, 0.54] | No |
| Yin et al. (2012) | 253 (66, 118, 69) | 18 wk | None | Classroom: 30-45 min (gross motor skills + dance) + 15-20 minutes (dance activities), daily; teacher training, 6 hr + 4 hr follow-up | BMIz: difference between control and center-based intervention only: B = −0.04; P > 0.1; difference between control and center-based + home intervention: −0.09; P < 0.09 Weight z-score: difference between control and center-based intervention only: B = −0.01; P > 0.1; difference between control and center-based + home intervention: −0.06; P < 0.04 | No |
| Zaragoza-Cortes et al. (2019) | 19 (10, 9) | NR | None | 7, 50 min sessions | Length-for-age z-score: no effect; P = 0.53 Weight-for-length z-score: no effect; P = 0.91 | No |

^*^ Hughes et al. 2020 and Hughes et al. 2021 report findings from the same intervention and study population at different time points.

| **Supplement 2.** Intervention and control/comparison condition description of the included studies (N = 40) | | | |
| --- | --- | --- | --- |
| Article | Intervention description | Theoretical basis | Control or comparison condition |
| Barkin et al. (2012) | Weekly 90-minute skills building sessions for parents and preschool-aged children designed to improve nutritional family habits, increase weekly physical activity, and decrease media use (sedentary activity). | Social cognitive theory and the transtheoretical model of change | Program based on the Dialogic Reading Model–C.A.R. (Comment and Wait, Ask Questions and Wait, and Respond by Adding More), an empirically tested curriculum that teaches parents to read picture books with their children. |
| Barkin et al. (2018) | Tiered-intensity program of decreasing intensity: (1) a 12-week intensive phase with weekly 90-minute skills-building sessions via either in-person groups or telephone calls; (2) a 9-month maintenance phase with monthly coaching telephone calls; and (3) a 24-month sustainability phase. Each week (intensive phase) or month (maintenance phase), participants created a self-defined goal about family health behaviors targeted in the intervention. The intervention included an adaptive component, an additional coaching telephone call that provided BMI results and additional guided goal setting and problem solving; this occurred when a child’s BMI category in-creased or remained obese at a data collection time point. | Social cognitive theory and the socioecological model | School-readiness program developed and delivered by the Nashville Public Library. The curriculum consisted of six 30-minute group-based activities delivered concurrently with data collection sessions. Participants in the intervention and control groups received the school-readiness program. |
| Bellows et al. (2013) | Weekly activities focused on a skill or group of skills from one of the three gross motor skill categories: stability (trunk strength), locomotor (running, hopping, skipping), or manipulation (ball skills). Food Friends characters were incorporated to introduce gross motor skills and movement concepts. Materials supporting the lessons, as well as creative themes and concepts, were provided as a kit for each intervention classroom Home connection materials and were sent home throughout the program. | NR | Both intervention and control classrooms implemented Food Friends, a 12-wk nutrition program shown to increase children’s willingness to try new foods. As part of Food Friends, children are introduced to Food Friends characters and learn about and taste new foods. |
| Berry et al. (2011) | Parent nutrition and exercise education, coping skills training, physical activity classes and Zumba dance classes. Children’s classes included Color Me Healthy for 45 minutes and then 1 hour of free time in the playground. Mothers were given examples of how to help their children increase their physical activity. | Social cognitive theory | Wait-list control |
| Bonuck et al. (2014) | Educational intervention delivered by WIC nutritionists delivered during WIC check-up. WIC nutritionists presented a flipchart with messages about healthy weight, dental caries, and iron deficiency anemia effects from bottle-weaning. It recommends that parents gradually replace bottles with cups. A pamphlet to share with family members and a lidded, two-handled 6-ounce sippy cup with a hard spout were given out at baseline. | NR | Standard WIC check-up |
| Cespedes et al. (2013) | Children received classroom activities, including Sesame Workshop Healthy Habits storybooks, posters, videos, games, and songs (1 hour daily); a “Healthy family day” workshop (1 hour); and weekly health notes. Parents participated in 3 workshops and weekly notes containing positive health messages about nutrition and active lifestyles to share with their children. Teachers participated in 3 centralized training sessions, plus personalized working sessions with a research supervisor (2 hours every 15 days) and received a teacher’s guide. | Social cognitive theory and the transtheoretical model in health promotion | Wait-list control |
| Chaparro et al. (2019) | This study assessed the impact of the 2009 WIC food package change. Changes included the addition of fruits, vegetables, and whole grains; reduction in the amount of juice, milk, cheese, and eggs offered; reductions in the fat levels allowed in milk; inclusion of culturally diverse replacement options; and reduction in the amount of formula for breastfeeding mothers. Children born after October 1, 2009 comprised the new package group. Children enrolled at birth - 4 years were classified as full-dose. Children enrolled between 2 and 3 years of age were late dose. | NR | Old package recipients included children who reached age 5 before October 2009 and were exposed to a full-dose (birth - 5 years of WIC enrollment) or half dose (enrolled between 2 and 3 years of age). |
| Cloutier et al. (2015) | 3–5-minute motivational interviewing sessions delivered to caregivers during sick or well child visits; a plan of action specific to the child's needs and one goal discussed during session; study toolkit containing a 6-oz cup, a measuring cup labeled with appropriate serving sizes for milk and juice, a portion size placemat, a foam ball (to encourage active play), and a pedometer (for caregiver); follow-up phone calls 5-7 days after each clinic visit. | Chronic care model | Usual care (height and weight measurements, calculation of BMI percentile by most clinicians, and counseling as deemed appropriate by the clinician). |
| Cloutier et al. (2018) | Standard Nurturing Families Network curriculum combined with education and enhanced support regarding breastfeeding; creation of a Family Wellness Plan that taught mothers goal-setting and self-monitoring skills; education and skill- building in behavioral strategies to implement desired changes in infant target areas; a toolkit with items to support the changes (e.g., 6 oz sippy cup, play mat); and linkages to community programs that support healthy behavior change including coupons for fruits and vegetables, cooking classes, exercise classes and breastfeeding support resources. | Chronic care model and the ecological model | Nurturing Families Network: home visitation program that uses an evidence-based curriculum (Parents as Teachers-Born to Learn, PAT). Curriculum focuses on the link between child development and parenting. Obesity prevention is not part of the PAT curriculum. |
| Costa et al. (2017) | Educational intervention on breast-feeding and complementary feeding based on the ‘Ten steps for the healthy feeding for Brazilian children from birth to two years of age'; information on proper food handling and hygiene; healthy meal pamphlet. | NR | Standard care |
| Crespo et al. (2012) | Home/Family environmental change (Fam-only): Promotora home visits, newsletters, recipe cards, goal setting, booster phone calls, goal setting, progress monitoring. Community-only environmental change (Comm-only): School playgrounds (improvements) and salad bars (implementation and improvement); community parks (improvements); Teachers’ discipline and classroom practices; physical education equipment, children’s menus at restaurants; posters, newsletters, frequent produce buyer cards in grocery stores. Family-plus-Community-environmental change (Fam+Comm): Fam-only + Comm-only interventions | Fam intervention: health belief model, social cognitive theory; Comm intervention: structural model of health behavior | Measurements only |
| Davis et al. (2016) | Head Start center curriculum focused on trying new fruits or vegetables and 30 min of physical activity daily; quarterly professional nutrition and physical activity development training for teachers and food service staff; food service component integrating policy and behavior change purchasing, preparation, and service; 2x per y family events and take-home materials about nutrition and physical activity; local grocery store component targeting store displays and food availability; healthcare provider component incorporating diet and physical activity messaging into routine patient visits. | Socioecological model | Standard Head Start curriculum |
| Fernandez-Jimenez et al. (2019) | Multicomponent, school-based, educational intervention targeting healthy diet, physical activity, bodily awareness and emotional regulation; involved parents and other family members in intervention activities, including FAMILIA days (informal and educational meetings) and 11 family-based health activities. | NR | Wait-list control |
| Fitzgibbon et al. (2013) | Children received a 3x/week intervention over 14 weeks. Sessions included nutrition instruction (activities led by puppets representing food groups) and 20 minutes of aerobic activity. Parents were encouraged to attend 6 weekly classes that included 60 minutes of interactive instruction on healthful eating and family exercise plus 30 minutes of moderate physical activity (e.g., salsa aerobics, walking group). Parents also received instruction on health risks associated with childhood obesity, the importance of modeling and reinforcing healthy eating and exercise patterns, and creation of a healthy home environment; weekly newsletters that paralleled the school-based component. | Health belief model and social cognitive theory | General Health Intervention: Children received a general health curriculum (e.g., dental health, seat belt safety, calling 911). Parents received weekly newsletters that paralleled the 14-week child curriculum. |
| French et al. (2018) | Primary Care + intervention program: home visiting, community-based parenting classes, and telephone check-in calls; referrals to community resources for healthy foods and physical activity opportunities. | Social ecological theory, motivational Interviewing | Primary Care program: providers discussed child BMI with parents at annual well-child visit, including messages about healthful eating and physical activity for the child; quarterly postcards focused on child development and school readiness. |
| Grummon et al. (2019) | Beverage intervention targeting environmental changes (increasing children’s access to water at child care centers and at home); policy changes (adoption and integration of the 2010 AB 2084, the Healthy Beverages in Childcare Policy passed in 2010 in CA); healthy beverage consumption at home; education activities for parents and children. | Social ecological framework | Delayed intervention |
| Haines et al. (2013) | Home-based intervention included motivational coaching by a health educator during 4 home visits and 4 health coaching telephone calls; mailed educational materials and incentives; weekly text messages on adoption of household routines. | Motivational Interviewing | Monthly mailed packages that included educational materials adapted from the CDC on reaching developmental milestones during early childhood and low-cost incentives (e.g., coloring books). |
| Haines et al. (2016) | Parent component: adaptation of the Chicago Parent Program to include lessons related to parental roles in promoting healthy nutrition and activity behaviors among children. Child component: sessions focused on a weight-related behavior addressed in the parent program with activities (reading a related book; exercise, preparing a healthful snack). | Social contextual framework | 9-week mailed educational materials on promoting healthy eating behaviors (e.g., My pyramid for preschoolers) |
| Heerman et al. (2019) | Two-phased intervention with in-person and maintenance phase. In-person phase: weekly sessions (90 min/session) targeting parent and child agency for health behaviors. Parent content included didactic and skills-building sessions on healthy diet, physical activity, sleep, engaged parenting, and media use. Children participated in a concurrent session mirroring adult intervention, then joined parents for activities and a shared meal. Parents received written materials to reinforce key messages from each session. Maintenance phase: bimonthly coaching calls over 3 months to set health goals, gauge progress on previous goals, and problem-solve. | Paradigm of personalized medicine; motivational interviewing; agency of change | Bi-monthly school readiness program |
| Hughes et al. (2020 and 2021)^*^ | Parents: 1/wk video-based sessions focused on child feeding practices followed by experiential learning activities. Topics included parental strategies to promote appropriate child portion sizes, structure and routines in the family environment, and dealing with outside influences on child eating. | Self-determination theory | Measurements only |
| Linville et al. (2020) | 8-session, 12 hr family-based group intervention targeting family-level health habits. Child and adult sessions were held separately. Parents were encouraged to develop SMART goals for their family to encourage change. | Cognitive dissonance theory, family systems theory, motivational interviewing, social ecological model | Families who reported English as the primary language spoken in the home were given a link to the video, “The Weight of the Nation,” a video about obesity in the U.S. Spanish-speaking families were sent a link to the video “Razones de Peso," about the obesity epidemic in Mexico. |
| Louzada et al. (2012) | Nutrition intervention that contained advice about breastfeeding and complementary feeding based on the “Ten steps for healthy feeding for Brazilian children from birth to 2 years of age” | NR | Usual well-baby care |
| Machuca et al. (2016) | The "Well Baby Group" (WBG) intervention was intended to be an alternative to traditional one-on-one well-child care. WBG visits are billable encounters incorporating all clinical components of routine well-child care. Model includes 11 sessions, adding three well-child care visits to the recommended American Academy of Pediatrics/Bright Futures schedule in the first year of life. Sessions (2-hr each) were nutrition education-focused and offered a venue to develop social networks. | Transtheoretical Model, Social Learning Theory, Freirian framework | Traditional, one-on-one well baby care |
| Martinez-Andrade et al. (2014) | Clinic-based intervention based on the High Five for Kids program. Educational sessions used motivational interviewing and reflexive listening techniques. Activities included real-world healthy lifestyle tasks such as playing active games, cooking healthy snacks, and creating shopping lists as a family. Sessions aimed to give families the tools to navigate health challenges and produce measurable changes. | Chronic Care Model | Usual clinic care - parents were informed of their child’s height and weight. Obese children were referred to a nutritionist if the physician considered it appropriate or given general dietary advice by the attending physician. |
| Natale et al. (2014) | Teacher component: Hip Hop curriculum + technical assistance to implement low fat, high fiber diet). Parent component: Eating Right Is Basic and Hip-Hop to Health Jr. programs. Parents were encouraged to reduce TV viewing, increase physical activity, and model healthy eating behaviors for their child at home. Specific components included a monthly educational dinner in which nutrition and physical activity were discussed, monthly newsletters, and at-home activities.  Center-Based modifications: development of policies to increase physical activity and healthy eating. A nutritionist worked with each child care center to modify menus to make them compliant with the policies and to ensure that the U.S. Department of Agriculture nutritional requirements were met. | Socioecological Framework | 1 visit from an injury prevention education mobile (ongoing program at the University of Miami). The mobile provided parents and teachers with hands-on safety education and information. |
| Natale et al. (2017) | Policy/environmental component: policies implemented in child-care centers based on the AAP Caring for Our Children (healthy drink, snack, PA and screen time policies). Parent and teacher role-modeling curriculum: training on effective role modeling of diet, food shopping, and ways to overcome barriers to healthy food choices. Program staff conducted joint parent–teacher meetings that focused on an evidence-based nutrition and PA curriculum.  Child curriculum: Lesson plans were designed to incorporate and reinforce the policy standards; half focused on beverage/snack policies and half focused on PA/screen time policies. | Social Cognitive Theory | Injury prevention curriculum delivered to 3 times to parents and teachers on home, car, and child seat safety information. |
| Palacios et al. (2018) | Short message service (SMS) intervention delivered to infant caregivers focused on proper infant feeding. Texts were designed to reinforce WIC messages on breastfeeding, preventing overfeeding, delaying introduction of solid foods, and delaying and reducing juice consumption. | Transtheoretical Model | SMS on general infant health topics (sleeping, bathing, teething, traveling in a car, medications, handling baby, and smoking, information related to immunization, and care of common illnesses) |
| Phelan et al. (2019) | Standard WIC care + internet-based weight loss program. Mothers had access to a website that incorporated weekly check-ins, pre-programmed and tailored feedback, behavioral video lessons, food diary, and group support features.  Texts: mother received 4 texts per week to support internet content. WIC sessions: monthly face-to-face group sessions offered at WIC. | NR | Standard WIC care |
| Romo et al. (2018) | Pilot intervention + home-based intervention support. Parents received an intervention training workshop, and workbooks to guide at-home activities. | Social Cognitive Theory | Pilot intervention: classroom-based physical activity and nutrition education curriculum using puppets, books, songs, structured motor activity and small incentives |
| Sadeghi et al. (2019) | Classroom component: science-based nutrition curricula aligned with the California state standards; Sport, Play and Active Recreation for Kids (SPARK) K-2 and Early Childhood (EC) PE curricula. Parent component: monthly "family nights" with discussions, a hand-on activity and food demonstration to reinforce obesity prevention messages. Economic component: monthly $25 fruit and vegetable voucher that allowed the same foods approved for the California Special Supplemental Nutrition Program for Women, Infants and Children Cash Value Voucher. | Social Learning Theory | Usual care |
| Salazar et al. (2014) | Preschool curriculum: newly developed national preschool education curriculum designed to include new standards for nutrition and physical activity.  Educator training: training in new curriculum and education provided to educators.  Parents: 1-2x/month “Healthy Days” activities for families to attend. Materials provided during activities focused on minimizing energy dense food, avoiding excess TV, and encouraging active play. | Socio-ecological Model | Usual preschool curriculum |
| Sangalli et al. (2021) | Medical staff: Physicians, nurses and administrative staff received a training session delivered by a nutritionist on the “Ten Steps for Healthy Feeding for Brazilian Children from Birth to Two Years of Age” from the Brazilian dietary guidelines for infants. Medical staff received leaflets about the guidelines to give to new mothers.  Mothers: Informational posters depicting how to introduce complementary foods and unhealthful foods for infants hung in waiting rooms during intervention. | NR | Usual care |
| Schwartz et al. (2015) | Standard Care + pro-breastfeeding counseling intervention. In the no-cohabitation group, adolescent mothers alone received the intervention. In the cohabitation group, both mother and grandmother received initial counseling. When mothers and grandmothers lived in the same household, joint counseling sessions were held. Sessions emphasized breastfeeding during the first 6 months after childbirth, and healthy complementary feeding starting at 6 months post-childbirth. Messages were reinforced with booklets and flipcharts designed specifically for the study. | NR | Standard maternity ward care |
| Sharma et al. (2019) | Preschool-based program modeled after CATCH (CATCH EC), targeting behavior through school environment modifications. CATCH EC has three main components: It’s Fun to be Healthy!, a nutrition and gardening-based curriculum; developmentally appropriate structured, indoor and outdoor PA; and parent tip-sheets including recipes, meal plans, parent-child activities, and recommendations for preschoolers’ diet, PA, and screen time. | Social Cognitive Theory | Wait-list control |
| Slusser et al. (2012) | 90-minute classes with content based on Bright Futures guidance, the “traffic Light” diet, the AAP expert work group’s recommendations, and the philosophy of internal regulation related to eating. | Social Learning Theory | Usual care, including standard WIC pamphlet typically given to patients at the VFC (waist-list control) |
| Taveras et al. (2021) | Staff training: standardized early-childhood obesity prevention training for pediatric clinicians and staff, including nurses, medical assistants, and WIC and home visiting staff.  Clinical services: enhanced tracking of infant weight gain, screening through clinical decision-support tools in the EHR, and surveillance by First 1000 Days health coaches; universal screening for adverse health behaviors and socio-contextual factors at ages 1, 6, and 12 months 25; educational materials and twice weekly text messaging to educate families on early-life health behaviors and to encourage behavior change.  Individual-level supports: patient navigation to support parents’ adoption of infant health behaviors, assess social needs, and strengthen integration of clinical and public health services (up to 3 phone calls from birth to 24 months); health coaching and care coordination (up to 3 phone calls from birth to 24 months) for women and infants considered to be high-risk for obesity based on pre pregnancy BMI, accelerated pregnancy weight gain, and/or rapid infant weight gain. | Social ecological model | Usual care |
| Washio et al. (2017) | Standard WIC care + economic incentives for breastfeeding. Participants were asked to demonstrate breastfeeding in front of research staff at monthly WIC or home sessions. Participants received $20 if breastfeeding was demonstrated at the end of the first month. Incentive amounts increased by $10 every month until the end of 6 months. When a participant did not demonstrate breastfeeding or pumping, she was given an opportunity to demonstrate breastfeeding within a week to be eligible for the monthly incentive. | Behavioral economics | Standard WIC breastfeeding services from WIC including on-site lactation consultation, bilingual peer counseling, weekly peer support meetings, free breast pump, and enhanced food package for breastfeeding mothers. When a participant had trouble with breastfeeding, she was referred to a bilingual home-visiting breastfeeding peer counselor in the area. |
| Yin et al. (2012) | Center-based intervention: Gross-motor skills intervention during daily 30-45 min outdoor play periods; classroom activities based on the Sesame Street Workshop Healthy Habits for Life resource kit and supplemented with nutrition and physical activity-themed activities; food tasting activities; parent newsletters, and staff wellness training. Center- + home-based intervention: center-based intervention + a short obesity prevention educational series delivered to parents during child pick-up time by peer educators. Education sessions took 5-10 minutes and used posters, worksheets, goody bags and a healthy snack to take home. | Systems approach | Usual preschool curriculum |
| Zaragoza-Cortes et al. (2019) | 7, 50-min sessions delivered to caregivers. Topics included complementary feeding, continued breastfeeding, accurate perception of child's weight, dietary diversity and nutrition for children 12 months of age and older. | NR | Measurements only until end of project, at which brief nutritional and MPCW orientation was given to this group. |

*Hughes et al. 2020 and Hughes et al. 2021 report findings from the same intervention and study population at different time points.

**Supplement 3. Overall risk of bias of the included studies, by domain**


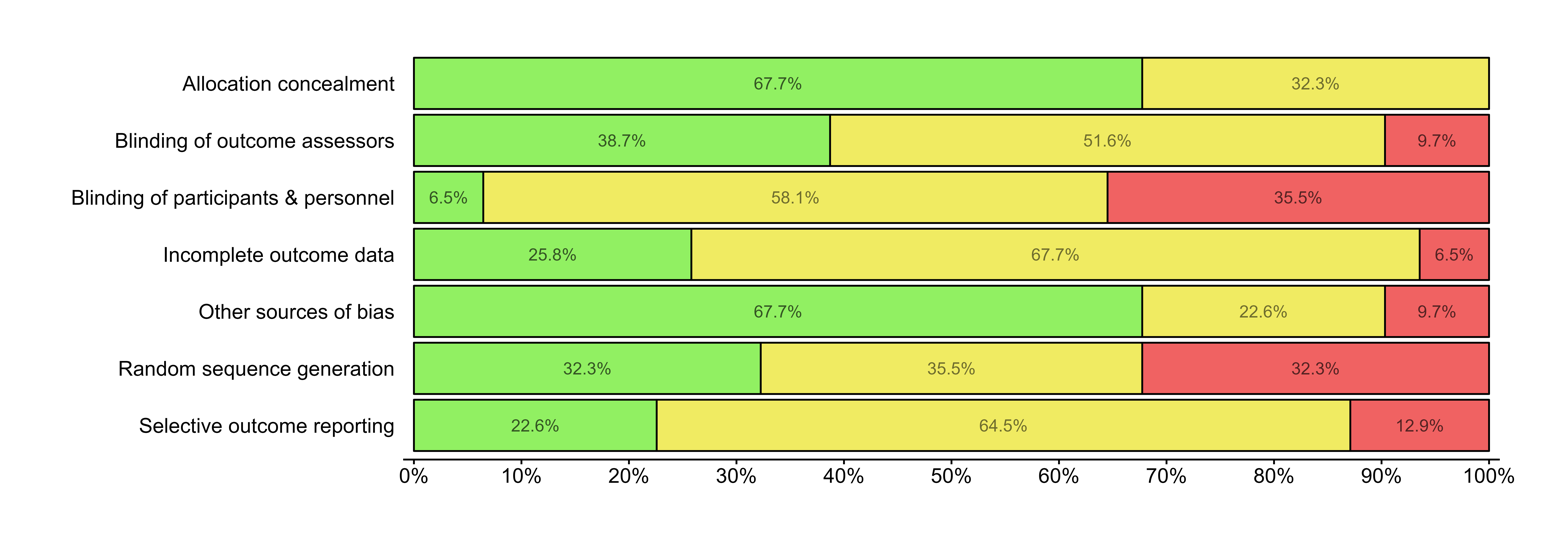


**Figure 3.1** Overall risk of bias by domain, randomized studies (n=30)

**
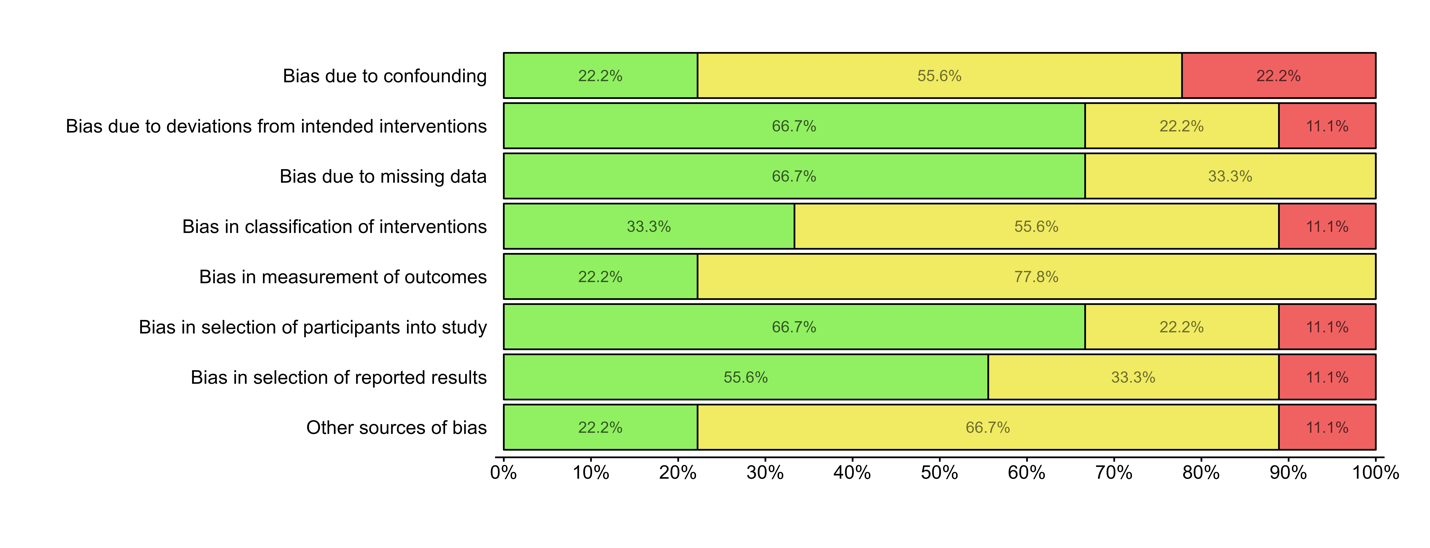
**

**Figure 3.2** Overall risk of bias by domain, non-randomized studies (n=9)

**Supplement 4. Risk of bias of the included studies**

**
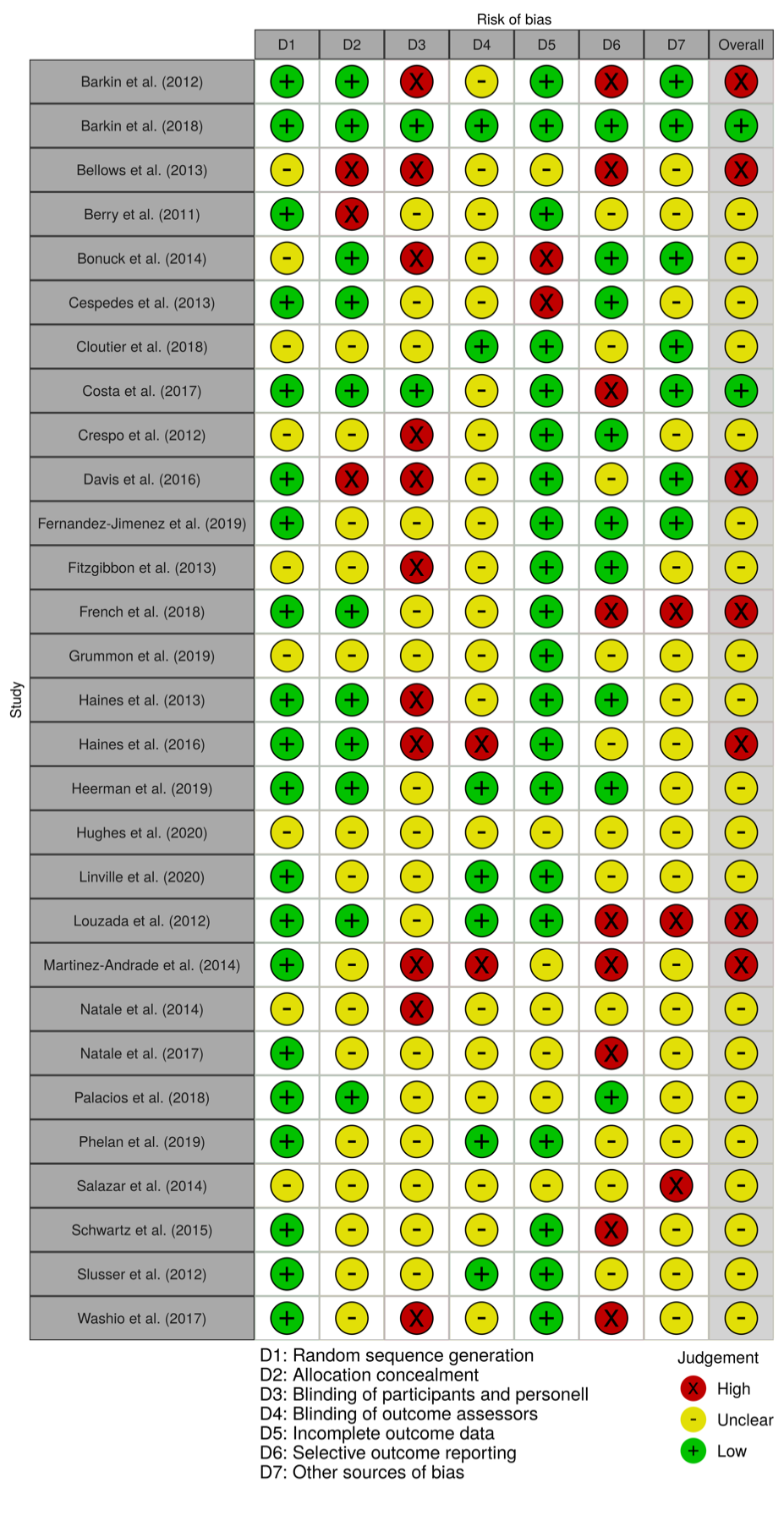

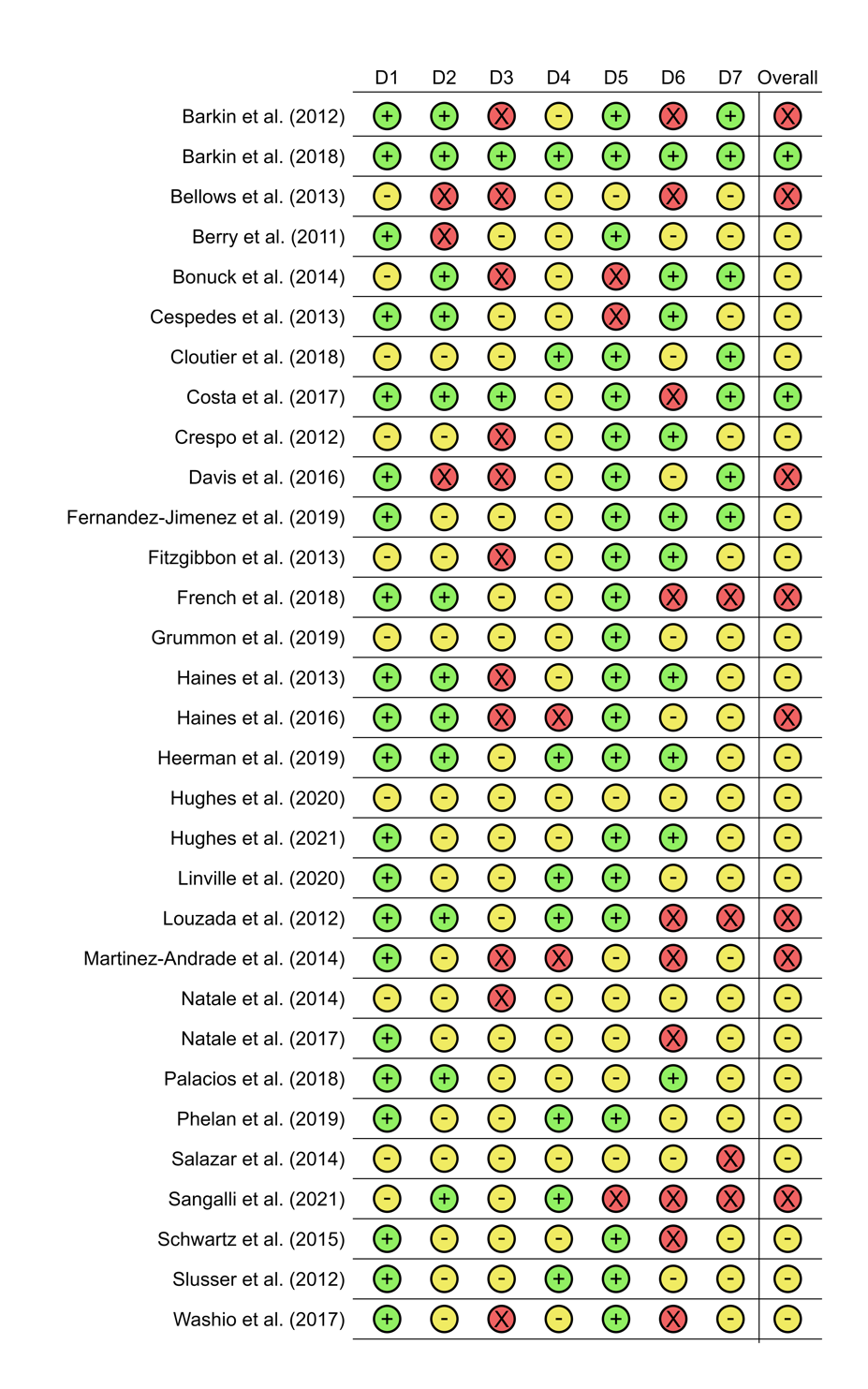
**

**Figure 4.1** Risk of bias judgements for individual domains, randomized studies (n=30)

**Figure 4.2** Risk of bias judgements for individual domains, non-randomized studies (n=9)

**
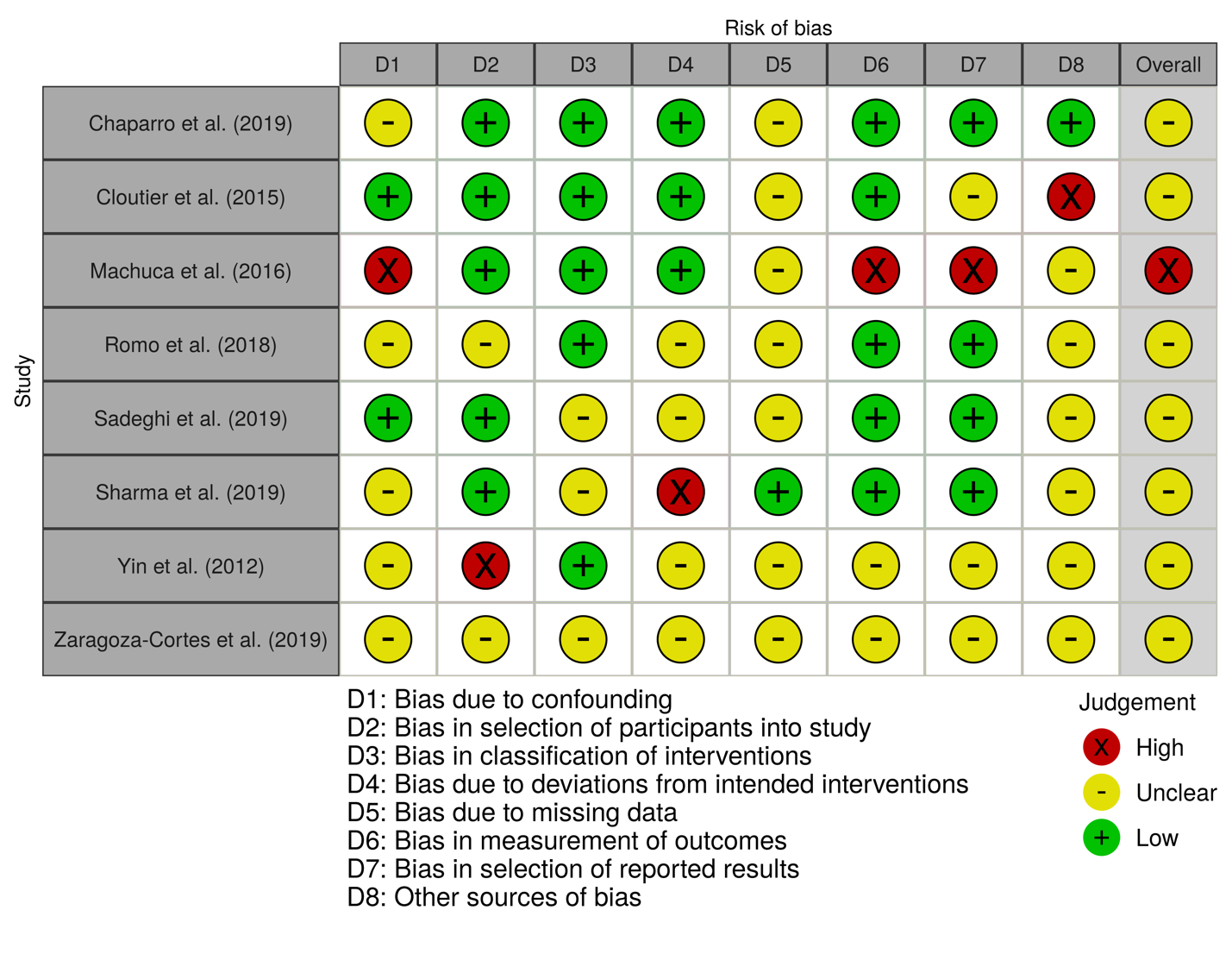

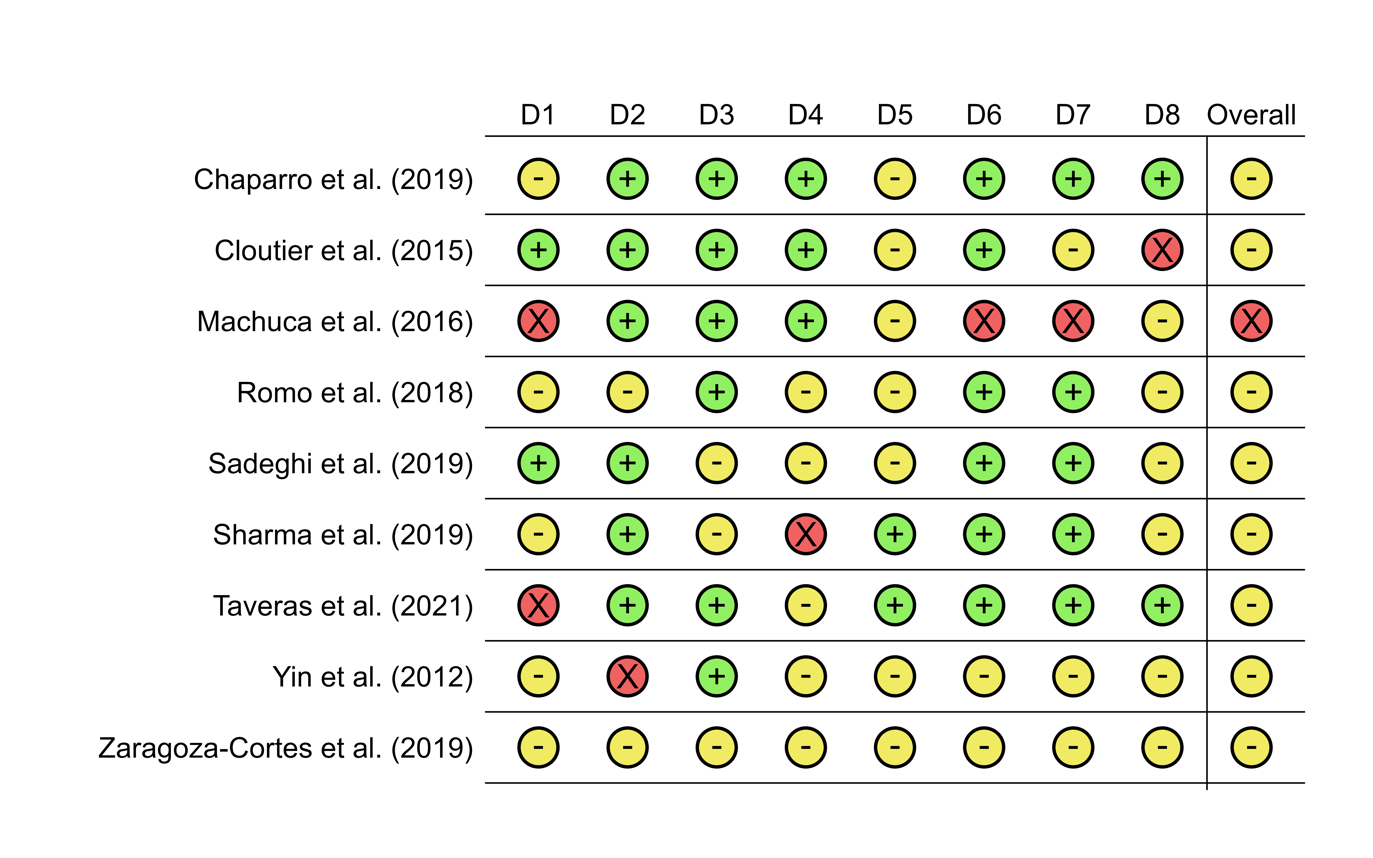
**

**APPENDICES**

| **Appendix 1.** Search strategy  *Sample search strategy for PubMed* | |
| --- | --- |
| #1 | intervention[Title/Abstract] OR approach*[Title/Abstract] OR strateg*[Title/Abstract] OR program*[Title/Abstract] OR trial[Title/Abstract] OR "program evaluation"[Title/Abstract] OR "prevention and control"[Subheading] OR "early intervention, educational"[MeSH] OR "randomized controlled trials as topic"[MeSH] OR "non randomized controlled trials as topic"[MeSH] OR "Controlled Clinical Trial"[Publication Type] OR "Evaluation Study"[Publication Type] OR "Randomized Controlled Trial"[Publication Type] |
| #2 | "behavior change"[Title/Abstract] OR “behavior modification”[Title/Abstract] OR behavior*[Title/Abstract] OR prevent*[Title/Abstract] OR "healthy eating"[Title/Abstract] OR "physical activity"[Title/Abstract] OR exercise[Title/Abstract] OR fitness[Title/Abstract] OR “health education"[MeSH] OR "Feeding Behavior"[MeSH] OR "Family Health"[MeSH] OR "Health Promotion"[MeSH] OR "Diet, Healthy"[MeSH] OR "Recreation"[MeSH] OR "Motor Activity"[MeSH] OR "Motor Skills"[MeSH] OR "Exercise"[MeSH] OR "screen time"[MeSH] OR "sleep"[MeSH] OR "Parents/education"[MeSH] |
| #3 | infan*[Title/Abstract] OR newborn*[Title/Abstract] OR baby[Title/Abstract] OR babies[Title/Abstract] OR pre-school*[Title/Abstract] OR preschool*[Title/Abstract] OR preschool-age*[Title/Abstract] OR "pre-school age*"[Title/Abstract] OR toddler*[Title/Abstract] OR child*[Title/Abstract] OR "nursery school*"[Title/Abstract] OR kinder*[Title/Abstract] OR pediatric[Title/Abstract] OR paediatric[Title/Abstract] OR afterschool[Title/Abstract] OR “infant"[MeSH] OR "Child, Preschool"[MeSH] OR "Child Day Care Centers"[MeSH] OR "Schools, Nursery"[MeSH] |
| #4 | obes*[Title/Abstract] OR overweight[Title/Abstract] OR "child* obesity"[Title/Abstract] OR BMI[Title/Abstract] OR "BMI z*"[Title/Abstract] OR "BMI-z*"[Title/Abstract] OR "body mass*"[Title/Abstract] OR "healthy weight"[Title/Abstract] OR "obesity prevention"[Title/Abstract] OR "weight status"[Title/Abstract] OR "weight-for-length"[Title/Abstract] OR "weight for length"[Title/Abstract] OR metaboli* [Title/Abstract] OR "Pediatric Obesity/prevention and control"[MeSH] OR "Pediatric Obesity/ethnology"[MeSH] OR "body weights and measures"[MeSH] OR "adiposity"[MeSH] OR "Body Mass Index"[MeSH] OR “Pediatric Obesity”[MeSH] OR "Overweight"[MeSH] OR "Overweight/ethnology"[MeSH] OR "Overweight/therapy"[MeSH] OR "Overweight/diagnosis"[MeSH] |
| #5 | Latin*[Title/Abstract] OR Hispan*[Title/Abstract] OR chican*[Title/Abstract] OR mexic*[Title/Abstract] OR divers*[Title/Abstract] OR “low*-income” [Title/Abstract] OR “low* income”[Title/Abstract] or urban[Title/Abstract] OR Spanish*[Title/Abstract] OR Portuguese*[Title/Abstract] OR "head start"[Title/Abstract] OR "early head start"[Title/Abstract] OR "Hispanic Americans"[MeSH] OR "Americas"[MeSH] OR "Socioeconomic Factors"[MeSH] OR "Minority Groups"[MeSH] OR "Urban Population"[MeSH] OR "Rural Population"[MeSH] OR "Poverty/ethnology"[MeSH] OR "Emigrants and Immigrants"[Mesh] |
| #6 | "Humans"[MeSH] |
| #7 | "Review"[Publication Type] OR "Meta-Analysis"[Publication Type] OR "Observational Study"[Publication Type] OR "Specialties, Surgical"[MeSH] OR "Pharmacology"[MeSH] OR "Cross-Sectional Studies"[MeSH] |
| #8 | #1 AND #2 AND #3 AND #4 AND #5 AND #6 NOT #7 |
| Limit | Published on or after January 1, 2010 |
|  |  |
| *Sample search strategy for Scopus* | |
| #1 | (TITLE-ABS-KEY ( intervention ) OR TITLE-ABS-KEY ( approach ) OR TITLE-ABS-KEY ( program* ) OR  TITLE-ABS-KEY ( strateg* )  OR  TITLE-ABS-KEY ("program evaluation") OR  TITLE-ABS-KEY ("controlled clinical trial") OR TITLE-ABS-KEY ("randomized controlled trial") OR TITLE-ABS-KEY ("non-randomized controlled trial") OR TITLE-ABS-KEY ("quasi-experimental") AND  DOCTYPE ( ar )  AND  PUBYEAR  >  2009 ) |
| #2 | (TITLE-ABS-KEY ("behavior change") OR TITLE-ABS-KEY ("behavior modification") OR TITLE-ABS-KEY (behavior*) OR TITLE-ABS-KEY (prevent*) OR TITLE-ABS-KEY ("healthy eating") OR TITLE-ABS-KEY ( "physical activity") OR TITLE-ABS-KEY (exercise) OR TITLE-ABS-KEY (fitness) OR TITLE-ABS-KEY ("health education") OR TITLE-ABS-KEY ("Feeding Behavior") OR TITLE-ABS-KEY ("Family Health") OR TITLE-ABS-KEY ("Health Promotion") OR TITLE-ABS-KEY ("healthy diet") OR TITLE-ABS-KEY ("Motor Activity") OR TITLE-ABS-KEY ("Motor Skills") OR TITLE-ABS-KEY ("screen time") OR TITLE-ABS-KEY (sleep) OR TITLE-ABS-KEY (parents) OR TITLE-ABS-KEY ("parent education") AND  DOCTYPE ( ar )  AND  PUBYEAR  >  2009 ) |
| #3 | (TITLE-ABS-KEY (infan*) OR TITLE-ABS-KEY (newborn*) OR TITLE-ABS-KEY (baby) OR TITLE-ABS-KEY (babies) OR TITLE-ABS-KEY (pre-school*) OR TITLE-ABS-KEY (preschool*) OR TITLE-ABS-KEY (preschool-age*) OR TITLE-ABS-KEY ("pre-school age*") OR TITLE-ABS-KEY (toddler*) OR TITLE-ABS-KEY ( child*) OR TITLE-ABS-KEY ("nursery school*") OR TITLE-ABS-KEY (kinder*) OR TITLE-ABS-KEY (pediatric) OR TITLE-ABS-KEY (paediatric) OR TITLE-ABS-KEY (afterschool) OR TITLE-ABS-KEY ("preschool child") OR TITLE-ABS-KEY ("child day care") AND DOCTYPE ( ar )  AND  PUBYEAR  >  2009 ) |
| #4 | (TITLE-ABS-KEY (obes*) OR TITLE-ABS-KEY (overweight) OR TITLE-ABS-KEY ("childhood obesity") OR TITLE-ABS-KEY (BMI) OR TITLE-ABS-KEY ("BMI z*") OR TITLE-ABS-KEY ("BMI-z*") OR TITLE-ABS-KEY ("body mass") OR TITLE-ABS-KEY ("body mass index") OR TITLE-ABS-KEY ("healthy weight") OR TITLE-ABS-KEY ("obesity prevention") OR TITLE-ABS-KEY ("weight status") OR TITLE-ABS-KEY ("weight-for-length") OR TITLE-ABS-KEY ("weight for length") OR TITLE-ABS-KEY (metaboli*) OR TITLE-ABS-KEY ("obesity prevention and control") OR TITLE-ABS-KEY ("adiposity") AND  DOCTYPE ( ar )  AND  PUBYEAR  >  2009 ) |
| #5 | (TITLE-ABS-KEY (Latin*) OR TITLE-ABS-KEY (mexic*) OR TITLE-ABS-KEY ("latin american*") OR TITLE-ABS-KEY (Hispanic) OR TITLE-ABS-KEY ("Hispanic American*") OR TITLE-ABS-KEY ( "Central America*") OR TITLE-ABS-KEY ("South America*") OR TITLE-ABS-KEY (caribbean) OR TITLE-ABS-KEY (chican*) OR TITLE-ABS-KEY (spanish*) OR TITLE-ABS-KEY ("spanish language") OR TITLE-ABS-KEY (portuguese) OR TITLE-ABS-KEY (dominican) OR TITLE-ABS-KEY (cuba*) OR TITLE-ABS-KEY ( belize* )  OR  TITLE-ABS-KEY ( "costa rica*" )  OR  TITLE-ABS-KEY ( "el  salvador*" )  OR  TITLE-ABS-KEY ( guatemala* )  OR  TITLE-ABS-KEY ( hondura* )  OR  TITLE-ABS-KEY ( nicaragua* )  OR  TITLE-ABS-KEY ( panama )  OR  TITLE-ABS-KEY ( argentin* )  OR  TITLE-ABS-KEY ( brazil* )  OR  TITLE-ABS-KEY ( paragu* )  OR  TITLE-ABS-KEY ( venezula* )  OR  TITLE-ABS-KEY ( surinam* )  OR  TITLE-ABS-KEY ( guyana* )  OR  TITLE-ABS-KEY ( columbia* ) OR  TITLE-ABS-KEY (peru*) OR TITLE-ABS-KEY (minorit*) OR TITLE-ABS-KEY ("minority group") OR TITLE-ABS-KEY (disadvantag*) OR TITLE-ABS-KEY ("low*-income") OR TITLE-ABS-KEY ("immigrant*") OR TITLE-ABS-KEY ("head start") OR TITLE-ABS-KEY ("early head start") OR TITLE-ABS-KEY ("urban population") OR TITLE-ABS-KEY ("rural population") OR TITLE-ABS-KEY ("poverty") OR TITLE-ABS-KEY ("social status") OR TITLE-ABS-KEY ("socioeconomic status") AND  DOCTYPE ( ar )  AND  PUBYEAR  >  2009 ) |
| #6 | (TITLE-ABS-KEY ("systematic review") OR TITLE-ABS-KEY ("cross-sectional study") OR TITLE-ABS-KEY ("prevalence study") OR TITLE-ABS-KEY ("drug therapy") OR TITLE-ABS-KEY (surgery) OR TITLE-ABS-KEY (surgical) OR TITLE-ABS-KEY ( pharmacolog*) ) |
| #7 | #1 AND #2 AND #3 AND #4 AND #5 AND NOT #6 |
| Limit | January 2010 - present |
| Limit | Article |

| **Appendix 2.** Studies excluded during full-text review, with reasons | | | |
| --- | --- | --- | --- |
| **Study** | **Title** | **Journal** | **Reason for Exclusion** |
| Kain 2010 | Effect of counselling school teachers on healthy lifestyle on the impact of a program to reduce childhood obesity | Revista Medica de Chile | Wrong child age |
| Saint-Maurice 2017 | Impact of NFL PLAY 60 Programming on Elementary School Children's Body Mass Index and Aerobic Capacity: The NFL PLAY 60 FitnessGram Partnership Project | J Sch Health | Wrong child age |
| Taveras 2017 | Clinical effectiveness of the Massachusetts childhood obesity research demonstration initiative among low-income children | Obesity (Silver Spring) | Wrong child age |
| Larsen 2017 | RE-AIM Analysis of a School-Based Nutrition Education Intervention in Kindergarteners | Journal of School Health | Wrong Outcomes |
| Cespedes 2013 | Promotion of cardiovascular health in preschool children: 36-month cohort follow-up | American Journal of Medicine | No Comparator |
| Cespedes 2012 | Opportunities to strengthen childhood obesity prevention in two Mexican health care settings | Int J Pers Cent Med | Wrong study design |
| Reifsnider 2018 | Randomized Controlled Trial to Prevent Infant Overweight in a High-Risk Population | Acad Pediatr | Wrong child age |
| Ordway 2018 | A home visiting parenting program and child obesity: A randomized trial | Pediatrics | Wrong child age |
| Navarro 2013 | The double task of preventing malnutrition and overweight: a quasi-experimental community-based trial | BMC Public Health | Wrong child age |
| Foster 2016 | A Pilot Study of Parent Mentors for Early Childhood Obesity | Journal of Obesity | Wrong exposure |
| Buscemi 2019 | Comparative Effectiveness Trial of an Obesity Prevention Intervention in EFNEP and SNAP-ED: Primary Outcomes | Nutrients | Not Latino/a or Hispanic (at least 50%) |
| Jastreboff 2018 | Preventing Childhood Obesity Through a Mindfulness-Based Parent Stress Intervention: A Randomized Pilot Study | J Pediatr | Not Latino/a or Hispanic (at least 50%) |
| Sanders 2014 | "Greenlight study ": A controlled trial of low-literacy, early childhood obesity prevention | Pediatrics | Methods or feasibility study |
| Lovera 2010 | Evaluation of a breastfeeding peer support program for fathers of Hispanic participants in a Texas special supplemental nutrition program for women, infants, and children | J Am Diet Assoc | Wrong outcome |
| WooBaidal 2017 | Childhood obesity prevention in the women, infants, and children program: Outcomes of the MA-CORD study | Obesity | Not Latino/a or Hispanic (at least 50%) |
| Rotheram-Fuller 2017 | Replicating Evidence-Based Practices with Flexibility for Perinatal Home Visiting by Paraprofessionals | Matern Child Health J | Exclusion reason: Wrong child age |
| Hawkins 2015 | A pregnancy lifestyle intervention to prevent gestational diabetes risk factors in overweight Hispanic women: A feasibility randomized controlled trial | Diabetic Medicine | Wrong child age |
| Trost 2011 | A nutrition and physical activity intervention for family child care homes | American Journal of Preventive Medicine | Wrong study design |
| Mihrshahi 2011 | Determinants of rapid weight gain during infancy: Baseline results from the NOURISH randomized controlled trial | BMC Pediatrics | Not Latino/a or Hispanic (at least 50%) |
| Taveras 2011 | First steps for mommy and me: A pilot intervention to improve nutrition and physical activity behaviors of postpartum mothers and their infants | Maternal and Child Health Journal | Not Latino/a or Hispanic (at least 50%) |
| Taveras 2015 | Comparative effectiveness of childhood obesity interventions in pediatric primary care: A cluster-randomized clinical trial | JAMA Pediatrics | Wrong child age |
| Teeters 2018 | Community readiness assessment for obesity research: Pilot implementation of the Healthier Families programme | Health Research Policy and Systems | Wrong study design |
| Treu 2017 | Advancing school and community engagement now for disease prevention (ASCEND): A quasi-experimental trial of school-based interventions to prevent childhood obesity | American Journal of Health Promotion | Wrong child age |
| van de Kolk 2019 | The effects of a comprehensive, integrated obesity prevention intervention approach (SuperFIT) on children's physical activity, sedentary behavior, and BMI z-score | International Journal of Environmental Research and Public Health | Not Latino/a or Hispanic (at least 50%) |
| Verbestel 2014 | Prevention of overweight in children younger than 2 years old: A pilot cluster-randomized controlled trial | Public Health Nutrition | Not Latino/a or Hispanic (at least 50%) |
| Yoong 2019 | The Impact of a Childcare Food Service Intervention on Child Dietary Intake in Care: An Exploratory Cluster Randomized Controlled Trial | American Journal of Health Promotion | Not Latino/a or Hispanic (at least 50%) |
| Wen 2019 | Linking two randomised controlled trials for Healthy Beginnings©: Optimising early obesity prevention programs for children under 3 years | BMC Public Health | Wrong study design |
| Sherwood 2015 | Pediatric Primary Care-Based Obesity Prevention for Parents of Preschool Children: A Pilot Study | Childhood Obesity | Not Latino/a or Hispanic (at least 50%) |
| Schwartz 2013 | Family-based risk reduction of obesity and metabolic syndrome: An overview and outcomes of the Idaho partnership for Hispanic health | Journal of Health Care for the Poor and Underserved | Wrong child age |
| Rodriguez-Ventura 2018 | A Comprehensive Intervention to Decrease Body Mass Index in Children with Adiposity: A Pilot Study | International Journal of Environmental Research and Public Health | Wrong child age |
| Rifas-Shiman 2017 | Two-year follow-up of a primary care-based intervention to prevent and manage childhood obesity: the High Five for Kids study | Pediatric Obesity | Not Latino/a or Hispanic (at least 50%) |
| Rhodes 2017 | A telephone intervention to achieve differentiation in dietary intake: a randomized trial in paediatric primary care | Pediatric Obesity | Wrong child age |
| Maguire 2010 | Office-based intervention to reduce bottle use among toddlers: TARget kids! Pragmatic, randomized trial | Pediatrics | Not Latino/a or Hispanic (at least 50%) |
| Peñalvo 2013 | The Program SI! intervention for enhancing a healthy lifestyle in preschoolers: First results from a cluster randomized trial | BMC Public Health | Not Latino/a or Hispanic (at least 50%) |
| Peñalvo 2013 | A cluster randomized trial to evaluate the efficacy of a school-based behavioral intervention for health promotion among children aged 3 to 5 | BMC Public Health | Wrong study design |
| Paul 2011 | Preventing obesity during infancy: A pilot study | Obesity | Not Latino/a or Hispanic (at least 50%) |
| Khanal 2015 | Effectiveness of a once per week delivery of a family-based childhood obesity intervention: A cluster randomised controlled trial | Pediatric Obesity | Wrong child age |
| Jiang 2019 | A community-based short message service intervention to improve mothers' feeding practices for obesity prevention: Quasi-experimental study | Journal of Medical Internet Research | Not Latino/a or Hispanic (at least 50%) |
| Hammersley 2019 | An internet-based childhood obesity prevention program (TIMe2bhealthy) for parents of preschool-aged children: Randomized controlled trial | Journal of Medical Internet Research | Not Latino/a or Hispanic (at least 50%) |
| Daniels 2015 | An early feeding practices intervention for obesity prevention | Pediatrics | Not Latino/a or Hispanic (at least 50%) |
| Daniels 2013 | Outcomes of an early feeding practices intervention to prevent childhood obesity | Pediatrics | Not Latino/a or Hispanic (at least 50%) |
| Daniels 2012 | Evaluation of an intervention to promote protective infant feeding practices to prevent childhood obesity: Outcomes of the NOURISH RCT at 14 months of age and 6 months post the first of two intervention modules | International Journal of Obesity | Not Latino/a or Hispanic (at least 50%) |
| Campbell 2013 | A parent-focused intervention to reduce infant obesity risk behaviors: A randomized trial | Pediatrics | Not Latino/a or Hispanic (at least 50%) |
| Breitenstein 2012 | The chicago parent program: Comparing 1-year outcomes for african american and latino parents of young children | Research in Nursing and Health | Not Latino/a or Hispanic (at least 50%) |
| Paz Soldán  2016 | Efecto de la estrategia “Chance de vida”; en la prevencia y tratamiento de ninos menores de cinco anos con sobrepeso u obesidad | Gaceta Medica Boliviana | Wrong study design |
| Oliveira 2011 | [Bolsa Familia Program and child nutritional status: strategic challenges] | Cien Saude Colet | Wrong exposure |
| Otterbach 2018 | Community-based childhood obesity prevention intervention for parents improves health behaviors and food parenting practices among Hispanic, low-income parents | BMC Obesity | No Comparator |
| Nabors 2015 | Implementation of an after-school obesity prevention program: helping young children toward improved health | Issues Compr Pediatr Nurs | Wrong child age |
| Martinez 2018 | Effects of a home-based participatory play intervention on infant and young child nutrition: A randomised evaluation among low-income households in El Alto, Bolivia | BMJ Global Health | Wrong outcome |
| Mareno 2014 | An early-phase translation study of the ways to enhance children's activity and nutrition (We Can!) programme for low-income families | Journal of Clinical Nursing | Wrong study design |
| Kronebusch 2019 | The impact of conditional cash transfers on nutrition outcomes: Experimental evidence from Mexico | Economics and Human Biology | Wrong child age |
| Lobos Fernandez 2013 | [Evaluation of an education intervention for childhood obesity prevention in basic schools in Chile] | Nutr Hosp | Wrong child age |
| Kobel 2019 | Intervention effects of a kindergarten-based health promotion programme on obesity related behavioural outcomes and BMI percentiles | Preventive Medicine Reports | Not Latino/a or Hispanic (at least 50%) |
| Esperat 2014 | TRANSFORMATION FOR HEALTH: OBESITY PREVENTION INTERVENTION AMONG HISPANIC CHILDREN IN WEST TEXAS | ANNALS OF BEHAVIORAL MEDICINE | No full text available |
| Elizondo-Montemayor 2014 | Individualized tailor-made dietetic intervention program at schools enhances eating behaviors and dietary habits in obese Hispanic children of low socioeconomic status | Scientific World Journal | Wrong child age |
| Peñalvo  2015 | The SI! Program for Cardiovascular Health Promotion in Early Childhood A Cluster-Randomized Trial | Journal of the American College of Cardiology | Not Latino/a or Hispanic (at least 50%) |
| Perez 2018 | Effectiveness of the bicisalud programme in a group of schoolchildren with excess weight | Pediatria de Atencion Primaria | Wrong child age |
| Po'e 2013 | Growing Right Onto Wellness (GROW): A family-centered, community-based obesity prevention randomized controlled trial for preschool child-parent pairs | Contemporary Clinical Trials | Wrong study design |
| Olson 2014 | The healthy start partnership: an approach to obesity prevention in young families | Fam Community Health | Wrong child age |
| Olsen 2012 | The healthy start project: A randomized, controlled intervention to prevent overweight among normal weight, preschool children at high risk of future overweight | BMC Public Health | Wrong study design |
| Okely 2020 | 'Jump start' childcare-based intervention to promote physical activity in pre-schoolers: six-month findings from a cluster randomised trial | The international journal of behavioral nutrition and physical activity | Not Latino/a or Hispanic (at least 50%) |
| Ogu 2014 | Hispanic Overweight and Obese Children: Thirty Cases Managed With Standard WIC Counseling or Motivational Interviewing | Infant, Child, and Adolescent Nutrition | Wrong study design |
| Novotny 2013 | University of Hawai'i Cancer Center Connection: The Pacific Way to Child Wellness: The Children's Healthy Living Program for Remote Underserved Minority Populations of the Pacific Region (CHL) | Hawaii J Med Public Health | Wrong study design |
| Nollen 2014 | Mobile technology for obesity prevention: A randomized pilot study in racial- and ethnic-minority girls | American Journal of Preventive Medicine | Wrong child age |
| Gunther 2017 | Simple Suppers: Child Findings of a Family Meals Childhood Obesity Prevention Intervention |  | Not peer-reviewed manuscript |
| Monsalves-Alvarez  2015 | Motor skills and nutritional status outcomes from a physical activity intervention in short breaks on preschool children conducted by their educators: a pilot study | Nutricion Hospitalaria | No Comparator |
| Narayanan 2019 | A school-based intervention using health mentors to address childhood obesity by strengthening school wellness policy | Preventing Chronic Disease | Wrong child age |
| Morales-Ruán  2014 | Effects of an intervention strategy for school children aimed at reducing overweight and obesity within the State of Mexico | Salud Publica Mex | Wrong child age |
| Lanigan 2016 | TrimTots: a healthy lifestyle intervention for prevention and treatment of obesity in preschool children: evidence from two randomised controlled trials: AB16 | Journal of Human Nutrition and Dietetics | Not peer-reviewed manuscript |
| Moran 2017 | Effect of a Lifestyle Intervention in Children with Obesity and Nonalcoholic Fatty Liver Disease | Topics in Clinical Nutrition | Wrong child age |
| Mazzeo 2012 | Stopping Childhood Obesity before It Begins | Phi Delta Kappan | Wrong study design |
| Manger 2012 | Obesity prevention in young schoolchildren: results of a pilot study | J Sch Health | Wrong child age |
| Madsen 2013 | School-community partnerships: A cluster-randomized trial of an after-school soccer program | JAMA Pediatrics | Wrong child age |
| Lima 2016 | Nutritional intervention with hypocaloric diet for weight control in children and adolescents with Prader-Willi Syndrome | Eating Behaviors | Wrong child age |
| Korenman 2013 | The Child and Adult Care Food Program and the Nutrition of Preschoolers | Early Childhood Research Quarterly | Not Latino/a or Hispanic (at least 50%) |
| Klish 2012 | Multicomponent school-initiated obesity intervention in a high-risk, Hispanic elementary school | J Pediatr Gastroenterol Nutr | Wrong child age |
| Kilanowski 2013 | Effects of a healthy eating intervention on Latina migrant farmworker mothers | Fam Community Health | Wrong child age |
| Kelly 2015 | The effect of a home-based strength training program on type 2 diabetes risk in obese Latino boys | Journal of Pediatric Endocrinology and Metabolism | Wrong child age |
| Keane 2011 | Effect of an obesity prevention intervention on fruit, vegetable and whole grain intake in American Indian and Hispanic preschoolers |  | Not peer-reviewed manuscript |
| Kain 2012 | [Evaluation of an obesity prevention intervention which included nutrition education and physical activity applied in public schools of Santiago, Chile] | Arch Latino am Nutr | Wrong child age |
| Johnson 2012 | Multilevel analysis of the Be Active Eat Well intervention: environmental and behavioural influences on reductions in child obesity risk | Int J Obes (Lond) | Wrong child age |
| Jelalian 2017 | Behavioral intervention in the treatment of obesity in children and adolescents: Implications for Mexico | Nutrition Reviews | Wrong study design |
| Hull 2018 | Childhood obesity prevention cluster randomized trial for Hispanic families: Outcomes of the healthy families study | Pediatric Obesity | Wrong child age |
| Hollar 2010 | Effect of a two-year obesity prevention intervention on percentile changes in body mass index and academic performance in low-income elementary school children | American Journal of Public Health | Wrong child age |
| Hollar 2010 | Effective multi-level, multi-sector, school-based obesity prevention programming improves weight, blood pressure, and academic performance, especially among low-income, minority children | J Health Care Poor Underserved | Wrong child age |
| Hoffman 2011 | Decaying behavioral effects in a randomized, multi-year fruit and vegetable intake intervention | Prev Med | Wrong child age |
| Hoffman 2012 | Farm to Family: Increasing Access to Affordable Fruits and Vegetables Among Urban Head Start Families | Journal of Hunger and Environmental Nutrition | Wrong study design |
| Berry 2016 | Results of the Optimizing Outcomes in Women with Gestational Diabetes Mellitus and Their Infants, a Cluster Randomized, Controlled Pilot Study: Lessons Learned | J Natl Black Nurses Assoc | Not Latino/a or Hispanic (at least 50%) |
| Weber 2014 | Lower protein content in infant formula reduces BMI and obesity risk at school age: Follow-up of a randomized trial | American Journal of Clinical Nutrition | Not Latino/a or Hispanic (at least 50%) |
| Vio 2014 | [Impact of a nutrition education intervention in teachers, preschool and basic school-age children in Valparaiso region in Chile] | Nutr Hosp | Wrong child age |
| Townsend 2012 | Baby knows best? The impact of weaning style on food preferences and body mass index in early childhood in a case-controlled sample | BMJ Open | Wrong study design |
| Tarro 2019 | Impact of a youth-led social marketing intervention run by adolescents to encourage healthy lifestyles among younger school peers (EYTO-Kids project): A parallel-cluster randomised controlled pilot study | Journal of Epidemiology and Community Health | Wrong child age |
| Suarez-Balcazar 2018 | Community-based participatory research to promote healthy lifestyles among Latino immigrant families with youth with disabilities | Scandinavian Journal of Occupational Therapy | Wrong study design |
| Sepúlveda  2019 | Feasibility, acceptability, and effectiveness of a multidisciplinary intervention in childhood obesity from primary care: Nutrition, physical activity, emotional regulation, and family | European Eating Disorders Review | Wrong child age |
| Sekhobo 2014 | Neighborhood disparities in prevalence of childhood obesity among low-income children before and after implementation of New York City child care regulations | Prev Chronic Dis | Wrong study design |
| Rushton 2015 | Enhancing child safety and well-being through pediatric group well-child care and home visitation: The Well Baby Plus Program | Child Abuse Negl | Wrong exposure |
| Rhyu 2019 | Effects of Telephone Follow-Up Intervention on %Body Fat, Inflammatory Cytokines, and Oxidative Stress in Obese Hispanic Children | Int J Environ Res Public Health | Wrong study design |
| Reyes-Morales 2016 | Effect of an intervention based on child-care centers to reduce risk behaviors for obesity in preschool children | Boletin Medico del Hospital Infantil de Mexico | Wrong outcome |
| Raynor 2012 | Efficacy of U.S. paediatric obesity primary care guidelines: two randomized trials | Pediatr Obes | Wrong study design |
| Parsons 2014 | Evaluating school wellness policy in curbing childhood obesity in Anchorage, Alaska | J Sch Nurs | Not Latino/a or Hispanic (at least 50%) |
| Heelan 2015 | Healthier School Environment Leads to Decreases in Childhood Obesity: The Kearney Nebraska Story | Child Obes | Wrong study design |
| Hawthorne 2011 | Grand Canyon Trekkers: School-based lunchtime walking program | The Journal of School Nursing | Not Latino/a or Hispanic (at least 50%) |
| Hammons 2019 | Effects of Culturally Tailored Nutrition Education on Dietary Quality of Hispanic Mothers: A Randomized Control Trial | Journal of Nutrition Education and Behavior | Wrong child age |
| Grutzmacher 2018 | Using text messages to engage low-income parents in school-based nutrition education | Journal of Hunger and Environmental Nutrition | Wrong study design |
| Frisvold 2011 | Expanding Exposure: Can Increasing the Daily Duration of Head Start Reduce Childhood Obesity? | Journal of Human Resources | Not peer-reviewed manuscript |
| Ford 2020 | Health outcomes of the Bolsa Família  program among Brazilian Amazonian children | Revista de Saude Publica | Wrong study design |
| Fagan 2010 | An obesity prevention program in a Delaware elementary school | Del Med J | Wrong study design |
| Espinoza 2017 | Effect of personal activity trackers on weight loss in families enrolled in a comprehensive behavioral family-lifestyle intervention program in the federally qualified health center setting: A randomized controlled trial | Contemporary Clinical Trials Communications | Wrong child age |
| Endevelt 2014 | An intensive family intervention clinic for reducing childhood obesity | Journal of the American Board of Family Medicine | Wrong child age |
| Polo-Oteyza 2017 | An intervention to promote physical activity in Mexican elementary school students: Building public policy to prevent noncommunicable diseases | Nutrition Reviews | Wrong child age |
| Preciado-Martinez 2016 | Effectiveness of sports teaching methods in the 40x40 program in overweight and obesity in school children | Revista Facultad de Medicina | Wrong child age |
| Resnicow 2015 | Motivational interviewing and dietary counseling for obesity in primary care: an RCT | Pediatrics | Not Latino/a or Hispanic (at least 50%) |
| Richmond 2014 | School programs and characteristics and their influence on student BMI: Findings from healthy passages | PLoS ONE | Wrong child age |
| Ríos-Cortázar  2013 | Reducing childhood obesity through nutrition component of a health promoting school initiative | Salud Publica de Mexico | Wrong child age |
| Rodríguez-Salinas Pérez  2018 | Effectiveness of the “Bicisalud” programme in a group of schoolchildren with excess weight | Pediatría Atención Primaria | Wrong child age |
| Sacher 2019 | Addressing childhood obesity in low-income, ethnically diverse families: Outcomes and peer effects of MEND 7-13  when delivered at scale in US communities | International Journal of Obesity | Wrong child age |
| Sadeghi 2017 | Multifaceted community-based intervention reduces rate of BMI growth in obese Mexican-origin boys | Pediatr Obes | Wrong child age |
| Saldiva 2010 | Anthropometric assessment and food intake of children younger than 5 years of age from a city in the semi-arid area of the Northeastern region of Brazil partially covered by the Bolsa Família program | Revista de Nutricao | Wrong study design |
| Schroeder 2015 | Early Obesity Prevention: A Randomized Trial of a Practice-Based Intervention in 0-24-Month Infants | J Obes | Not Latino/a or Hispanic (at least 50%) |
| Schuler 2019 | Building Blocks for Healthy Children: Evaluation of a Child Care Center-Based Obesity Prevention Pilot Among Low-Income Children  Among Low-Income Children | Journal of Nutrition Education and Behavior | Not Latino/a or Hispanic (at least 50%) |
| Scruzzi 2014 | School health: An educational intervention on nutrition from an integrated approach | Cuadernos.info | Wrong child age |
| Shamah-Levy 2017 | Effects of SaludArte program in feeding and nutrition components in school children in Mexico City | Salud Publica de Mexico | Wrong child age |
| Siegel 2014 | Obese children in a community YMCA "Fun 2B Fit" program have a reduction in BMI Z-scores | Clin Pediatr (Phila) | Wrong child age |
| Silva 2011 | Overweight and obesity in five- to ten-year-old children benefited from Bolsa Família Program in the State of Sergipe, Brazil | Revista Paulista de Pediatria | Wrong study design |
| Sorkin 2014 | Dyadic collaboration in shared health behavior change: The effects of a randomized trial to test a lifestyle intervention for high-risk Latinas | Health Psychology | Wrong child age |
| Sosa 2016 | ¡Miranos! (Look at Us! We Are Healthy!) : Home-Based and Parent Peer-Led Childhood Obesity Prevention | Health Promotion Practice | Wrong outcome |
| Haemer 2013 | A clinical model of obesity treatment is more effective in preschoolers and Spanish speaking families | Obesity | Wrong study design |
| Haire-Joshu 2018 | A Randomized Trial of Weight Change in a National Home Visiting Program | Am J Prev Med | Wrong child age |
| dosSantos 2015 | Nutritional condition of children who benefit from the " Bolsa Família " programme in a city of northwestern São Paulo State, Brazil | Journal of Human Growth and Development | Wrong study design |
| Diaz Martinez  2015 | Eficacia de un programa de actividad fisica y alimentacion saludable en escolares chilenos | Hacia la PromociÃ³n de la Salud | Wrong child age |
| Bonvecchio-Arenas 2010 | The school as an opportunity for obesity prevention: An experience from the Mexican school system | Revista Espanola de Nutricion Comunitaria | Wrong outcome |
| Davis 2016 | Outcomes of a weight management program conjointly addressing parent and child health | Children's Health Care | Wrong child age |
| Souto-Gallardo 2020 | Association of Food Parenting Practices on Child BMI z Score and Waist Circumference in Mexican Preschool Children After 1 Year of Follow-Up | Journal of Nutrition Education and Behavior | Wrong study design |
| Subica 2016 | Communities of color creating healthy environments to combat childhood obesity | American Journal of Public Health | Wrong study design |
| Taveras 2011 | Randomized controlled trial to improve primary care to prevent and manage childhood obesity: the High Five for Kids study | Arch Pediatr Adolesc Med | Not Latino/a or Hispanic (at least 50%) |
| Taverno Ross 2018 | ANDALE Pittsburgh: results of a promotora-led, home-based intervention to promote a healthy weight in Latino preschool children | BMC Public Health | No Comparator |
| Telford 2013 | Physical education can improve insulin resistance: The LOOK randomized cluster trial | Medicine and Science in Sports and Exercise | Wrong child age |
| Tomayko 2017 | Evaluation of a multi-year policy-focused intervention to increase physical activity and related behaviors in lower-resourced early care and education settings: Active Early 2.0 | Preventive Medicine Reports | Not Latino/a or Hispanic (at least 50%) |
| Toussaint 2017 | Slowing BMI Growth Trajectories in Elementary School-Aged Children: The Northeast Iowa Food and Fitness Initiative | Fam Community Health | Not Latino/a or Hispanic (at least 50%) |
| Tripicchio 2018 | Clinical-community collaboration: A strategy to improve retention and outcomes in low-income minority youth in family-based obesity treatment | Childhood Obesity | Wrong child age |
| Vicedo 2012 | Extracurricular school interventions aimed at the effects of physical activity on 1st of eso children with overweight and obesity | Revista de Psicologia del Deporte | Wrong child age |
| Vilchis-Gil 2016 | Decreased Body Mass Index in Schoolchildren After Yearlong Information Sessions With Parents Reinforced With Web and Mobile Phone Resources: Community Trial | J Med Internet Res | Wrong child age |
| Vilchis-Gil 2018 | Effect on the Metabolic Biomarkers in Schoolchildren After a Comprehensive Intervention Using Electronic Media and In-Person Sessions to Change Lifestyles: Community Trial | J Med Internet Res | Wrong child age |
| Villar 2020 | Padres Activos (Active Fathers): An Experiential Learning Approach to Obesity Prevention and Health Engagement among Latino Fathers and Their Children | Journal of Latinos and Education | Wrong study design |
| Warschburger 2018 | Evaluation of an approach avoidance training intervention for children and adolescents with obesity: A randomized placebo-controlled prospective trial | European Eating Disorders Review | Wrong child age |
| Van Horn 2018 | Dietary Approaches to Stop Hypertension Diet and Activity to Limit Gestational Weight: Maternal Offspring Metabolics Family Intervention Trial, a Technology Enhanced Randomized Trial | Am J Prev Med | Wrong child age |
| Ziebarth 2012 | A community-based family intervention program to improve obesity in Hispanic families | Wmj | Wrong child age |
| Wen 2012 | Effectiveness of home-based early intervention on children's BMI at age 2: Randomised controlled trial | BMJ (Online) | Not Latino/a or Hispanic (at least 50%) |
| Wen 2015 | Sustainability of effects of an early childhood obesity prevention trial over time: A further 3-year follow-up of the healthy beginnings trial | JAMA Pediatrics | Not Latino/a or Hispanic (at least 50%) |
| Xu 2017 | A Community-Based Nutrition and Physical Activity Intervention for Children Who Are Overweight or Obese and Their Caregivers | J Obes | Wrong child age |
| Ye 2019 | Effects of school-based exergaming on urban children’s physical activity and cardiorespiratory fitness: A quasi-experimental study | International Journal of Environmental Research and Public Health | Wrong child age |
| Yun 2015 | A randomized, home-based, childhood obesity intervention delivered by patient navigators Health behavior | BMC Public Health | Wrong study design |
| Tucker 2019 | Evaluation of a Primary Care Weight Management Program in Children Aged 2(-)5 years: Changes in Feeding Practices, Health Behaviors, and Body Mass Index | Nutrients | Not Latino/a or Hispanic (at least 50%) |
| Sharma 2018 | Pilot evaluation of HEAL â€“ A natural experiment to promote obesity prevention behaviors among low-income pregnant women | Preventive Medicine Reports | Not Latino/a or Hispanic (at least 50%) |
| Schwartz 2016 | Effect of a School-Based Water Intervention on Child Body Mass Index and Obesity | JAMA Pediatr | Not Latino/a or Hispanic (at least 50%) |
| Schaefer 2015 | Assessing child obesity and physical activity in a hard-to-reach population in California’s Central Valley, 2012-2013 | Preventing Chronic Disease: Public Health Research, Practice, and Policy | Not Latino/a or Hispanic (at least 50%) |
| Rodriguez 2015 | Evaluating impacts of school-based extension garden programs from a child's perspective | Journal of Extension | Wrong study design |
| Paul 2011 | ENERGIZE! A community-based lifestyle intervention targeting at-risk, overweight children | N C Med J | Not Latino/a or Hispanic (at least 50%) |
| Paul 2018 | Effect of a Responsive Parenting Educational Intervention on Childhood Weight Outcomes at 3 Years of Age: The INSIGHT Randomized Clinical Trial | Jama | Not Latino/a or Hispanic (at least 50%) |
| Ostbye 2012 | Parent-focused change to prevent obesity in preschoolers: results from the KAN-DO study | Prev Med | Not Latino/a or Hispanic (at least 50%) |
| Nemet 2011 | Health promotion intervention in low socioeconomic kindergarten children | The Journal of Pediatrics | Not Latino/a or Hispanic (at least 50%) |
| Nelson 2018 | Racial/ethnic differences in the effectiveness of a multisector childhood obesity prevention intervention | American Journal of Public Health | Not Latino/a or Hispanic (at least 50%) |
| Haines 2018 | Guelph Family Health Study's Home-Based Obesity Prevention Intervention Increases Fibre and Fruit Intake in Preschool-Aged Children | Canadian Journal of Dietetic Practice and Research | Not Latino/a or Hispanic (at least 50%) |
| Jensen 2015 | Effectiveness of a Parent Health Report Intervention to Increase Physical Activity among Preschoolers and Kindergarteners | Children's Health Care | Not Latino/a or Hispanic (at least 50%) |
| Hoelscher 2010 | Reductions in child obesity among disadvantaged school children with community involvement: The Travis County CATCH Trial | Obesity | Wrong child age |
| Hodgkinson 2019 | An educational intervention to prevent overweight in pre-school years: a cluster randomised trial with a focus on disadvantaged families | BMC Public Health | Not Latino/a or Hispanic (at least 50%) |
| Hilgendorf 2016 | Lessons from a pilot community-driven approach for obesity prevention | Wisconsin Medical Journal | Wrong study design |
| Herazo-Beltrán  2018 | Programme’s effect of physical activity in the abdominal perimeter and body mass index in schools | Revista Latinoamericana de Hipertension | Wrong child age |
| Herman 2012 | "Eat Healthy, Stay Active!": a coordinated intervention to improve nutrition and physical activity among Head Start parents, staff, and children | Am J Health Promot | Not Latino/a or Hispanic (at least 50%) |
| Hayes 2019 | Home and neighbourhood built environment features in family-based treatment for childhood obesity | Pediatric Obesity | Wrong child age |
| Hatfield 2017 | Impact of a community-based physical activity program on fitness and adiposity among overweight and obese children | Health Promotion Practice | Wrong child age |
| Hannon 2019 | Implementation and Evaluation of the Abriendo Caminos Program: A Randomized Control Trial Intervention for Hispanic Children and Families | Journal of Nutrition Education and Behavior | Wrong study design |
| Haire-Joshu 2015 | A group randomized controlled trial integrating obesity prevention and control for postpartum adolescents in a home visiting program | International Journal of Behavioral Nutrition and Physical Activity | Wrong child age |
| Hacke 2019 | Effectiveness of a physical activity intervention in preschoolers: A cluster-randomized controlled trial | Scand J Med Sci Sports | Not Latino/a or Hispanic (at least 50%) |
| Gross 2016 | Randomized Controlled Trial of a Primary Care-Based Child Obesity Prevention Intervention on Infant Feeding Practices | J Pediatr | Wrong child age |
| Grills 2014 | Communities Creating Healthy Environments: improving access to healthy foods and safe places to play in communities of color | Prev Med | Wrong study design |
| Greenhouse 2014 | Prescription for Parks: A Creative Approach to Combat Childhood Obesity in South Carolina and To Get South Carolina Children Moving | J S C Med Assoc | Wrong study design |
| George 2016 | Healthy Lifestyle Fitness Camp: A Summer Approach to Prevent Obesity in Low-Income Youth | J Nutr Educ Behav | Wrong child age |
| Garza 2019 | Effects of telephone aftercare intervention for obese Hispanic children on body fat percentage, physical fitness, and blood lipid profiles | International Journal of Environmental Research and Public Health | Wrong child age |
| Foster 2019 | A pilot study of low-income mothers and fathers of preschool age children to determine the relationship of food security and nutrition assistance on feeding style and child body weight | Journal of Hunger and Environmental Nutrition | Not Latino/a or Hispanic (at least 50%) |
| Gold 2020 | Ripple Effects of the Communities Preventing Childhood Obesity Project | Health Promotion Practice | Wrong study design |
| Falbe 2015 | Active and Healthy Families: A Randomized Controlled Trial of a Culturally Tailored Obesity Intervention for Latino Children | Acad Pediatr | Wrong child age |
| Early 2019 | Motivational interviewing and home visits to improve health behaviors and reduce childhood obesity: A pilot study | Hispanic Health Care International | Wrong child age |
| Duggins 2010 | Impact of family YMCA membership on childhood obesity: a randomized controlled effectiveness trial | J Am Board Fam Med | Wrong child age |
| De la Haye 2019 | In-home obesity prevention in low-income infants through maternal and social transmission | Contemporary Clinical Trials | Wrong study design |
| Davison 2013 | A childhood obesity intervention developed by families for families: Results from a pilot study | The International Journal of Behavioral Nutrition and Physical Activity | Not Latino/a or Hispanic (at least 50%) |
| Feng 2016 | A Multicomponent Intervention Helped Reduce Sugar-Sweetened Beverage Intake in Economically Disadvantaged Hispanic Children | Am J Health Promot | Wrong child age |
| Davis 2011 | The use of TeleMedicine in the treatment of paediatric obesity: feasibility and acceptability | Matern Child Nutr | Wrong child age |
| Davis 2013 | Outcomes from an urban pediatric obesity program targeting minority youth: the Healthy Hawks program | Child Obes | Wrong child age |
| Cespedes 2014 | Participant characteristics and intervention processes associated with reductions in television viewing in the High Five for Kids study | Preventive Medicine: An International Journal Devoted to Practice and Theory | Not Latino/a or Hispanic (at least 50%) |
| DaSilva 2013 | The effectiveness of a physical activity and nutrition education program in the prevention of overweight in schoolchildren in Criciúma, Brazil | European Journal of Clinical Nutrition | Wrong child age |
| Cruz 2016 | Effects of an Obesity Prevention Intervention on Physical Activity Among Preschool Children: The CHILE Study | Health Promot Pract | Wrong outcome |
| Crespo 2018 | A randomized controlled trial to prevent obesity among Latino paediatric patients | Pediatric Obesity | Wrong child age |
| Costa-Urrutia 2019 | Effect of multi-component school-based program on body mass index, cardiovascular and diabetes risks in a multi-ethnic study | BMC Pediatrics | Wrong child age |
| Braungart-Rieker 2014 | Psychosocial pathways to childhood obesity: A pilot study involving a high-risk preschool sample | Eating Behaviors | Not Latino/a or Hispanic (at least 50%) |
| Bird 2019 | SANOS Y FUERTES: HEALTHY & STRONG: BEHAVIORAL OUTCOMES FROM AN OBESITY PREVENTION INTERVENTION FOR LATINO FAMILIES | ANNALS OF BEHAVIORAL MEDICINE | Not peer-reviewed manuscript |
| Eichen 2020 | Impact of Race and Ethnicity on Weight-Loss Outcomes in Pediatric Family-Based Obesity Treatment | Journal of Racial and Ethnic Health Disparities | Wrong child age |
| Elder 2014 | Childhood obesity prevention and control in city recreation centres and family homes: the MOVE/me Muevo Project | Pediatr Obes | Wrong child age |
| Engelbert 2015 | Utilization of the RE-AIM framework (Reach, Effectiveness, Adoption, Implementation, and Maintenance) to assess outcomes of an obesity prevention intervention in the preschool setting | 8th Annual Conference on the Science of Dissemination and Implementation | Not peer-reviewed manuscript |
| DeBock 2012 | Positive impact of a pre-school-based nutritional intervention on children's fruit and vegetable intake: Results of a cluster-randomized trial | Public Health Nutrition | Not Latino/a or Hispanic (at least 50%) |
| Deavenport-Saman 2019 | Early Childhood Obesity Among Underserved Families: A Multilevel Community-Academic Partnership | Am J Public Health | Wrong study design |
| Dooyema 2013 | The childhood obesity research demonstration project: a comprehensive community approach to reduce childhood obesity | Child Obes | Wrong study design |
| Duncanson 2013 | Effect of a low-intensity parent-focused nutrition intervention on dietary intake of 2- to 5-year olds | Journal of Pediatric Gastroenterology and Nutrition | Not Latino/a or Hispanic (at least 50%) |
| Ek 2019 | A parent treatment program for preschoolers with obesity: A randomized controlled trial | Pediatrics | Not Latino/a or Hispanic (at least 50%) |
| Correa 2010 | CAN DO Houston: a community-based approach to preventing childhood obesity | Prev Chronic Dis | Wrong study design |
| Derose 2019 | A Community-Partnered Approach to Developing Church-Based Interventions to Reduce Health Disparities Among African-Americans and Latinos | Journal of Racial and Ethnic Health Disparities | Wrong study design |
| Domoff 2018 | Parent-child interaction therapy as a prevention model for childhood obesity: A novel application for high-risk families | Children and Youth Services Review | Wrong study design |
| Cohen 2014 | Preventing chronic disease through improving food and activity environments | Child Obes | Wrong study design |
| Cluss 2010 | Adapting pediatric obesity treatment delivery for low-income families: a public-private partnership | Clin Pediatr (Phila) | Wrong child age |
| Chen 2014 | Cooking up diversity. Impact of a multicomponent, multicultural, experiential intervention on food and cooking behaviors among elementary-school students from low-income ethnically diverse families | Appetite | Not Latino/a or Hispanic (at least 50%) |
| Bryars 2011 | The Effects of a School Based Obesity Prevention/intervention Program Targeting Preschool Children |  | No Comparator |
| Buchan 2010 | The influence of a high intensity physical activity intervention on a selection of health related outcomes: An ecological approach | BMC Public Health | Wrong study design |
| Carroll 2011 | Overcoming barriers to vegetable consumption by preschool children: A child care center buying club | Journal of Hunger and Environmental Nutrition | Wrong study design |
| Brotman 2012 | Early childhood family intervention and long-term obesity prevention among high-risk minority youth | Pediatrics | Wrong child age |
| Castro 2013 | Growing Healthy Kids: A community garden-based obesity prevention program | American Journal of Preventive Medicine | Wrong child age |
| Castaneda 2017 | Obesity and Diabetes: Evaluating the Impact of Promotores as a Prevention and Intervention Strategy in the Latino Community |  | Not peer-reviewed manuscript |
| Burrows 2013 | Pilot intervention in an economically disadvantaged community: The back-to-basics after-school healthy lifestyle program | Nutrition and Dietetics | Wrong child age |
| BÃ¼rgi 2012 | Effect of a lifestyle intervention on adiposity and fitness in socially disadvantaged subgroups of preschoolers: A cluster-randomized trial (Ballabeina) | Preventive Medicine: An International Journal Devoted to Practice and Theory | Not Latino/a or Hispanic (at least 50%) |
| Buonomo 2019 | Access to health care, nutrition and dietary habits among school-age children living in socio-economic inequality contexts: results from the “ForGood: Sport is Well-Being” programme | International Journal of Food Sciences and Nutrition | Wrong child age |
| Bruno 2019 | HomeStyles Express: a telephone counseling-based childhood obesity prevention intervention for parents of preschool-aged children |  | Not peer-reviewed manuscript |
| Bruno 2018 | Home-exercise Childhood Obesity Intervention: A Randomized Clinical Trial Comparing Print Versus Web-based (Move It) Platforms | J Pediatr Nurs | Wrong child age |
| Bories 2013 | A rural, noncompetitive youth running program that aims to make a difference | Childhood Obesity | Wrong child age |
| Bohnert 2014 | Active summers matter: evaluation of a community-based summertime program targeting obesogenic behaviors of low-income, ethnic minority girls | New directions for youth development | Wrong child age |
| Bohnert 2017 | Improving Urban Minority Girls™ Health Via Community Summer Programming | Journal of Racial and Ethnic Health Disparities | Wrong child age |
| Biggs 2020 | Identifying Opportunities to Promote Physical Activity in a Diverse Low-Income Population: A Mixed-Method Study at a Boys &amp; Girls Club Site | Child and Youth Care Forum | Wrong child age |
| Berry 2014 | The family partners for health study: A cluster randomized controlled trial for child and parent weight management | Nutrition and Diabetes | Wrong child age |
| Berry 2017 | A cluster randomized controlled trial for child and parent weight management: Children and parents randomized to the intervention group have correlated changes in adiposity | BMC Obesity | Wrong child age |
| Benjamin-Neelon 2015 | A community-based intervention increases physical activity and reduces obesity in school-age children in North Carolina | Child Obes | Wrong child age |
| Baker 2012 | Fitter, healthier, happier families: A partnership to treat childhood obesity in the West Midlands | Public Health | Wrong child age |
| Annesi 2016 | Effects of an after-school care-administered physical activity and nutrition protocol on body mass index, fitness levels, and targeted psychological factors in 5- to 8-year-olds | Transl Behav Med | Wrong child age |
| Andersen 2015 | Participation in the Juntos Conditional Cash Transfer Program in Peru Is Associated with Changes in Child Anthropometric Status but Not Language Development or School Achievement1-4 | Journal of Nutrition | Wrong child age |
| Alkon 2014 | Nutrition and physical activity randomized control trial in child care centers improves knowledge, policies, and children's body mass index | BMC Public Health | Wrong child age |
| Brito 2014 | Effects of a diabetes prevention programme on weight-specific quality of life in Latino youth | Pediatric Obesity | Wrong child age |
| Briones-Villalba 2018 | Effect of a physical activity program and nutritional education to prevent sugar sweetened beverages consumption and development of obesity among schoolchildren in Tijuana, Mexico | Revista Espanola de Nutricion Humana y Dietetica | Wrong child age |
| Bowen 2018 | Changing the housing environment to reduce obesity in public housing residents: A cluster randomized trial | BMC Public Health | Wrong child age |
| Boles 2015 | Influencing the Home Food and Activity Environment of Families of Preschool Children Receiving Home-Based Treatment for Obesity | Clin Pediatr (Phila) | Wrong study design |
| Blanck 2015 | The Childhood Obesity Research Demonstration project: linking public health initiatives and primary care interventions community-wide to prevent and reduce childhood obesity | Child Obes | Wrong study design |
| Bird 2015 | Sanos y Fuertes: An Obesity Prevention Intervention for Latino Families | Journal of Nutrition Education and Behavior | Not peer-reviewed manuscript |
| Berger-Jenkins 2014 | Evaluation of a Coordinated School-Based Obesity Prevention Program in a Hispanic Community: Choosing Healthy and Active Lifestyles for Kids/Healthy Schools Healthy Families | American Journal of Health Education | Wrong child age |
| Bender 2013 | A culturally appropriate intervention to improve health behaviors in Hispanic mother-child dyads | Child Obes | No Comparator |
| Belansky 2010 | Early effects of the federally mandated local wellness policy on school nutrition environments appear modest in Colorado's rural, low-income elementary schools | Journal of the American Dietetic Association | Not Latino/a or Hispanic (at least 50%) |
| Barbosa Filho 2017 | For whom was it effective? Moderators of the effect of a school-based intervention on potential physical activity determinants among Brazilian students | Preventive Medicine | Wrong child age |
| McClaskey 2010 | A childhood obesity program in federally qualified community health centers | J Health Care Poor Underserved | Wrong study design |
| Lu 2018 | Four months of a school-based exercise program improved aerobic fitness and clinical outcomes in a Low-SES population of normal weight and overweight/obese children with asthma | Frontiers in Pediatrics | Wrong child age |
| Long 2020 | Evaluation of a pragmatic trial of a collaborative school-based obesity prevention intervention in a low-income urban district | Preventive Medicine | Wrong child age |
| Lau 2013 | Effects of an occupation-based obesity prevention program for children at risk | Occupational Therapy In Health Care | Wrong child age |
| Landry 2019 | Cooking and gardening behaviors and improvements in dietary intake in hispanic/latino youth | Childhood Obesity | Wrong child age |
| Holm 2012 | Parental influence on child change in physical activity during a family-based intervention for child weight gain prevention | J Phys Act Health | Wrong child age |
| Herbst 2019 | Effective Implementation of Culturally Appropriate Tools in Addressing Overweight and Obesity in an Urban Underserved Early Childhood Population in Pediatric Primary Care | Clin Pediatr (Phila) | Not Latino/a or Hispanic (at least 50%) |
| de Heer 2017 | Let’s Move Together: A Randomized Trial of the Impact of Family Health History on Encouragement and Co-Engagement in Physical Activity of Mexican-Origin Parents and Their Children | Health Education and Behavior | Wrong child age |
| Mennella 2018 | Type of infant formula increases early weight gain and impacts energy balance: a randomized controlled trial | Am J Clin Nutr | Not Latino/a or Hispanic (at least 50%) |
| McCarron 2010 | Shaping America's Youth initiative: implementation and assessment of a community-based approach to improving childhood nutrition and physical activity. Introduction | Pediatrics | Wrong study design |
| Kong 2010 | A pilot walking school bus program to prevent obesity in Hispanic elementary school children: role of physician involvement with the school community | Clin Pediatr (Phila) | No Comparator |
| Landaeta-Jimenez 2013 | Programa de educacion nutricional en escuelas de tres ciudades en Venezuela | Anales Venezolanos de Nutricion | No Comparator |
| Anderson 2014 | A randomized pilot trial of an integrated school-worksite weight control program | Health Psychol | Not Latino/a or Hispanic (at least 50%) |
| Anderson 2015 | Taking steps together: A family- and community-based obesity intervention for urban, multiethnic children | Health Education & Behavior | Wrong child age |
| Alvirde-Garcia 2013 | [Results of a community-based life style intervention program for children] | Salud Publica Mex | Wrong child age |

| **Appendix 3.** Risk of bias assessment for randomized studies by outcome (n = 40) | | | | | | |  |
| --- | --- | --- | --- | --- | --- | --- | --- |
| Article | Number analyzed | Outcome(s) | Detailed effect | Effect (positive, no effect, negative) | Risk of Bias | Explanation |  |
| Barkin et al. (2012) | 75 | BMI | B = –0.59 [95% CI: –0.94 to –0.25]; P = .001 | Positive | High | Not blinded; unclear if assessors were blinded; obese sub-analysis not pre-specified |  |
| Barkin et al. (2018) | 610 | BMI (short-term) | 36 mo, adjusted: B = 0.05 [95% CI, −0.29 to 0.38]; P = .79 | No effect | Low | Double-blinded study; high retention rate; all outcomes reported in NCT registry |  |
|  | 610 | BMI (long-term) | 3-yr: no effect (P = .39) | No effect | Low |  |  |
|  | 610 | Risk of OB | 3-mo post-intervention follow-up, adjusted: RR = 0.51 [95% CI, 0.29- 0.92]; P = .02; corrected P = .10 | No effect | Low |  |  |
| Bellows et al. (2013) | 201 | BMI | No effect (regression results NR) | No effect | Unclear | Blinding of outcome assessors not reported; no trial registry |  |
|  | 201 | BMIz | No effect (regression results NR) | No effect | Unclear |  |  |
| Berry et al. (2011) | 56 | BMI % | Intervention (baseline, follow-up): 86 ± 7.5, 82 ± 6.8 Control: 86 ± 11.1, 88 ± 8.5; P for difference = .03 | Positive | Unclear | Pilot study; research staff not concealed; blinding not detailed; high retention; no NCT; methods poorly reported |  |
| Bonuck et al. (2014) | 135 | Weight-for-length > 85th % | OR = 1.02 [95% CI = 0.5-2.0]; P = .95 | No effect | High | Sequence generation and blinding unclear; not masked; high attrition rate |  |
| Cespedes et al. (2013) | 858 | BMI (short-term) | 6-mo: Intervention (baseline, follow-up): 15.99 ± 0.09, 16.57 ± 0.45; Control: 15.77 ± 0.08, 16.40 ± 0.49; P for difference = .19 | No effect | Unclear | Blinding not reported; high attrition rate |  |
|  |  | BMI (long-term) | 18-mo: no effect (P = 0.5) | No effect | Unclear |  |  |
| Cloutier et al. (2018) | 34 | Weight-for-length BMI (short term) | 6-mo: 17.5 ± 1.5, 16.9 ± 2.0, P for difference = .37 | No effect | High | Outcome not prespecified in trial registry |  |
|  |  | Weight-for-length BMI (long term) | 12-mo: 17.4 ± 1.6, 17.3 ± 1.0, P for difference = .83 | No effect | High |  |  |
|  |  | Weight-for-length z-score (short term) | 6-mo: 0.4 ± 1.1, 0.9 ± 2.7, P for difference = .61 | No effect | High |  |  |
|  |  | Weight-for-length z-score (long term) | 12-mo: 0.2 ± 1.2, 0.8 ± 2.4, P for difference = .51 | No effect | High |  |  |
| Costa et al. (2017) | 396 | BMIz (birth - 1 yr) | Girls: 0.11 [95% CI, −0.31, 0.53]; P = .61 Boys: −0.07 [95% CI, −0.40, 0.27]; P = .69 | No effect | High | Outcome not prespecified in trial registry |  |
|  | 345 | BMIz (1-4 yr) | Girls: − 0.01 [95% CI, − 0.32, 0.30]; P = .94 Boys: −0.07 [95% CI, −0.41, 0.27]; P = .67 | No effect | High |  |  |
|  | 305 | BMIz (4-8 yr) | Girls: 0.15 [95% CI, −0.14, 0.45]; P = .32 Boys: −0.29 [95% CI, −0.65, 0.06]; P = .10 | No effect | High |  |  |
| Crespo et al. (2012) | 441 | BMIz | Comm main effect: B = −0.19, SE = 0.33; P = .54; Fam main effect: .003, SE = 0.33; P = .92 | No effect | Unclear | Participants and promotoras unblinded; methods poorly reported; unclear if control participated in other interventions |  |
|  |  | BMI % | Comm main effect: B = 0.20, SE = 0.84; P = .72; Fam main effect: B = .72, SE = 0.85; P = .40 | No effect | Unclear |  |  |
|  |  | Weight status | Comm main effect: B = −0.19, SE = 0.29; P = .53; Fam main effect: B = .036, SE = 0.29; P = .90 | No effect | Unclear |  |  |
| Davis et al. (2016) | 1,816 | BMIz | change per 6 month study time: intervention slope = 0.039 [95% CI, 0.014 to 0.063]; comparison slope = 0.038 [95% CI, 0.014, 0.063; difference = 0.011 [95% CI −0.024, 0.046]; P = 0.54 | No effect | High | Study design precluded blinding to intervention status; trial registry not reported |  |
| Fernandez-Jimenez et al. (2019) | 448 | BMIz | B = −0.03 [95% CI, −0.29, 0.24]; P = .84 | No effect | Unclear | Blinding not reported |  |
| Fitzgibbon et al. (2013) | 128 | BMI (short-term) | B = −0.04 [95% CI, −0.13, 0.05]; P value NR | No effect | Unclear | Poorly reported sequence generation, concealment and blinding; no description of treatment fidelity; unclear if control participated in other interventions |  |
|  |  | BMI (long-term) | B = 0.22 [95% CI, 0.02, 0.41]; P < 0.05 | Negative | Unclear |  |  |
|  |  | BMIz (short term) | B = −0.03 [95% CI, −0.13, 0.06]; P value NR | No effect | Unclear |  |  |
|  |  | BMIz (long-term) | −0.03 [95% CI, −0.03, 0.17]; P value NR | No effect | Unclear |  |  |
| French et al. (2018) | 534 | BMI | B = –0.19 [95% CI, –0.64, 0.26] | No effect | Unclear | Blinding not reported; low treatment fidelity |  |
|  |  | BMIz | B = –0.07 [95% CI, –0.23, 0.08] | No effect | Unclear |  |  |
|  |  | BMI % | B = –0.33 [95% CI, –3.93, 3.26] | No effect | Unclear |  |  |
|  |  | Waist circumference (cm) | B = –0.58 [95% CI, –1.91, 0.76] | No effect | Unclear |  |  |
|  |  | Triceps skinfold (cm) | B = –0.35 [95% CI, –1.62, 0.92] | No effect | Unclear |  |  |
| Grummon et al. (2019) | 154 | Prevalence of OW/OB | Mean = −6% [95% CI, −15%, 3%] | No effect | Unclear | Unclear sequence generation and randomization, trial registry unavailable; unclear if control participated in other interventions |  |
|  |  | BMI % | 1.2 [95% CI, −2.5, 4.9] | No effect | Unclear |  |  |
|  |  | BMI | 0.03 [95% CI, −0.2, 0.3] | No effect | Unclear |  |  |
| Haines et al. (2013) | 111 | BMI | B = −0.40 [95% CI, −0.79, 0.00]; P = .05 | Positive | Unclear | Participant blinding not possible due to study design; staff blinding not reported; unclear if control participated in other interventions |  |
|  |  | BMIz | B = −0.17 [95% CI, −0.40, 0.07]; P = .17 | No effect | Unclear |  |  |
| Haines et al. (2016) | 94 | BMI (short-term) | B = −0.36 [95% CI, −1.23, 0.51); P = 0.41 | No effect | High | Non-blinded; inclusion criteria unclear; does not state whether participants could participate in other interventions |  |
|  | 96 | BMI (long-term) | B = 0.18 [95% CI, −0.58, 0.93); P = 0.64 | No effect | High |  |  |
|  | 94 | BMIz (short term) | B = −0.25 [95% CI, −0.75, 0.25); P = 0.33 | No effect | High |  |  |
|  | 96 | BMIz (long-term) | B = 0.06 [95% CI, −0.36, 0.48); P = 0.64 | No effect | High |  |  |
| Heerman et al. (2019) | 117 | BMI | B = –0.41 [95% CI: –0.82 to 0.01]; P = .05 | Positive | Low | BMI and BMI trajectory specified in trial registry |  |
| Hughes et al. (2020) | 178 | BMIz | No effect (P = 0.8) | No effect | Unclear | Methods poorly reported |  |
| Hughes et al. (2021) | 135 | BMIz | No effect (P = 0.91) | No effect | Unclear | Methods poorly reported |  |
| Linville et al. (2020) | 27 | BMI | B = 0.035; SE - 0.114; P = .758 | No effect | Unclear | Allocation concealment and participant blinding unclear; some methods poorly reported; pilot study |  |
| Louzada et al. (2012) | 396 | Risk of OW (12-16 mo) | Boys: RR = 0.95 [95% CI: 0.65, 1.39] Girls: RR = 0.99 [95% CI: 0.68, 1.43] | No effect | High | Participants not blinded; high retention rate; follow-up analyses not specified in original NCT; home visits may have influenced results |  |
|  |  | Risk of OB (12-16 mo) | Boys: RR = 0.84 [95% CI: 0.31, 2.22] Girls: RR = 1.24 [95% CI: 0.55, 2.77] | No effect | High |  |  |
|  | 344 | Risk of OW (3-4 yr) | Boys: RR = 1.05 [95% CI: 0.58, 1.89] Girls: RR = 1.23 [95% CI: 0.68, 2.19] | No effect | High |  |  |
|  |  | Risk of OB (3-4 yr) | Boys: RR = 1.31 [95% CI: 0.33, 5.09] Girls: RR = 3.04 [95% CI: 0.81, 11.31] | No effect | High |  |  |
|  | 308 | Risk of OW (7-8 yr) | Boys: RR = 1.20 [95% CI: 0.75, 1.91] Girls: RR = 1.19 [95% CI: 0.67, 2.10] | No effect | High |  |  |
|  |  | Risk of OB (7-8 mo) | Boys: RR = 1.73 [95% CI: 0.77, 3.90] Girls: RR = 1.24 [95% CI: 0.47, 3.22] | No effect | High |  |  |
| Martinez-Andrade et al. (2014) | 101 | BMI (short-term) | 3 mo: B = 0.23 [95% CI: –0.07, 0.54] | No effect | High | Staff not blinded at follow-up assessments; low retention, but used multiple imputation to retain sample size |  |
|  | 108 | BMI (long-term) | 6 mo: B = 0.06 [95% CI: –0.17, 0.23] | No effect | High |  |  |
| Natale et al. (2014) | 239 | BMIz (short term) | 6 mo: intervention: B = 0.57 [95% CI: 0.24, 0.91]; control: B = 0.25 [95% CI: −0.41, 0.91] | No effect | Unclear | Poorly detailed methods; study not blinded; low retention for BMI analyses; no NCT; intervention fidelity not reported |  |
|  | 185 | BMIz (long-term) | 12 mo: B = 0.72 [95% CI: 0.42, 1.03); B = 0.76 [95% CI: 0.16, 1.35]; P for difference = 0.81 | No effect | Unclear |  |  |
| Natale et al. (2017) | 1211 | BMIz | B = –1.95, SE = 0.97; P = 0.04 | Positive | Unclear | Attrition rate not reported; blinding not reported |  |
| Palacios et al. (2018) | 170 | Weight-for-age z-score | intervention: −0.05 (0.85); control: −0.05 (0.85); P for difference = 0.976 | No effect | Unclear | Unclear blinding; attrition rate varied by ethnicity; high retention rate |  |
|  |  | Length-for-age z-score | intervention: 0.59 (1.57); control: 0.61 (1.56); P for difference = 0.931 | No effect | Unclear |  |  |
|  |  | BMIz | intervention: −0.53 (1.38); control: −0.54 (1.39); P for difference = 0.958 | No effect | Unclear |  |  |
| Phelan et al. (2019) | 296 | BMIz | B = 0.19 [95% CI: −0.08, 0.46]; P = 0.16 | No effect | Unclear | Unclear concealment; results poorly reported |  |
|  |  | Triceps skinfold for age z-score | B = −0.04 [95% CI: −0.42, 0.35]; P = 0.85 | No effect | Unclear |  |  |
|  |  | Subcapular skinfold for age z-score | B = −0.30 [95% CI: −0.71, 0.11]; P = 0.16 | No effect | Unclear |  |  |
| Sangalli et al. (2021) | 715 | Waist circumference | Primary follow-up timepoint NR | No effect | High | Unclear allocation concealment and blinding; incomplete outcome data reporting; selective reporting on timepoints not specified in NCT |  |
|  |  | Triceps skinfold thickness | Primary follow-up timepoint NR | No effect |  |  |  |
|  |  | Subcapular skinfold thickness | Primary follow-up timepoint NR | No effect |  |  |  |
| Salazar et al. (2015) | 265 | Body fat % | OB: intervention, -1.5 ± 1.8; control, 1.3 ± 2.0; P < 0.01  Normal weight: intervention, -0.7 ± 2.2; control, 1.0 ± 2.1; P < 0.01 | Positive | Unclear | Results poorly reported; randomization not described; did not report on relevant covariates |  |
|  |  | Weight-for-height z-score | OB: intervention, -0.1 ± 0.5; control, -0.1 ± 0.7; P = .64  Normal weight: intervention, -0.02 ± 0.4; control, -0.05 ± 0.4; P = 0.56 | No effect | Unclear |  |  |
| Schwartz et al. (2015) | 207 | Prevalence of OW/OB | RR = 1.24 [95% CI: 0.86, 1.81]; P = 0.254 | No effect | High | Unclear allocation concealment and blinding; weight outcome not specified a-priori |  |
| Slusser et al. (2012) | 121 | BMIz | Mean difference between intervention and control: −0.24, SE = 0.11; P = 0.04 | Positive | Unclear | Allocation concealment and blinding unclear |  |
|  |  |  |  |  |  |  |  |
|  |  |  |  |  |  |  |  |
| Washio et al. (2017) | 35 | Weight-for-age z-score | B = −0.14 [95% CI: –0.81, 0.54] | No effect | High | Unclear allocation concealment; not blinded; 12-month outcomes not reported; possible cross-contamination effect of WIC participation |  |

| **Appendix 4.** Risk of bias assessment for non-randomized studies by outcome (n = 9) | | | | | | |
| --- | --- | --- | --- | --- | --- | --- |
| Article | Number analyzed | Outcome(s) | Effect | Outcome (positive, no effect, negative) | Risk of Bias | Explanation |
| Chaparro et al. (2019) | 57,171 | Weight-for-height z-score | Figure only | No effect | Unclear | Unclear if WIC participants participated in other obesity prevention programs; number of excluded participants not reported |
|  |  | BMIz | Figure only | No effect | Unclear |  |
|  |  | Risk of OB | Boys: RR = 0.88 [95% CI, 0.86 - 0.91]; P < .05 Girls: RR = 0.90 [95% CI, 0.87 - 0.93]; P < .05 | Positive | Unclear |  |
| Cloutier et al. (2015) | 418 | BMI % | B = −0.23 [95% CI, −0.33, −0.13] | Positive | Unclear | Rates of attrition and reasons for attrition not reported; unclear if participants participated in other programs; weight subgroup analysis nor specified a-priori |
| Machuca et al. (2016) | 187 | Prevalence of OW/OB | OR = 0.12 [95% CI: 0.02, 0.94] | Positive | High | Intervention group breastfed for 1 month longer on average; did not examine differences in outcome by breastfeeding duration; retention not reported; outcomes measured at variable times |
| Romo et al. (2018) | 276 | BMIz | B = −0.28 [95% CI: −0.17, −0.40; P < 0.001 | Positive | Unclear | Unable to adjust for important confounders; fidelity not reported; differential loss to follow-up |
| Sadeghi et al. (2019) | 603 | BMIz | Girls: B = 0.30; SE = 0.12; P = 0.01 Boys: B = 0.14; SE = 0.15; P = 0.36 | Positive (girls only) | Unclear | Concealment and blinding unclear, uneven treatment dose |
|  |  | BMI | Girls: B = 2.70; SE = 0.02; P < 0.001 Boys: B = 2.69; SE = 0.02; P < 0.001 | Positive | Unclear |  |
|  |  | Waist circumference to height ratio | Girls: B = 0.48; SE = 0.01; P < 0.001 Boys: B = 0.47; SE = 0.01; P < 0.001 | Positive | Unclear |  |
| Sharma et al. (2019) | 848 | BMIz | B = −0.26 [95% CI: −0.50, −0.01]; P = 0.041 | Positive | Unclear | Possible confounding due to Head Start center merger; intervention elements implemented in some control centers |
|  |  | BMI % | B = −6.5 [95% CI: −12.4, −0.69]; P = 0.028 | Positive | Unclear |  |
| Taveras et al. (2021) | 1837 | Weight-for-length z-score | B = −0.27 [95% CI: –0.39, –0.15] | Positive | Unclear | Implementation fidelity not reported; missing data not accounted for in analyses |
|  |  | Odds of overweight | OR = 0.46 [95% CI: 0.28, 0.76] | Positive | Unclear |  |
| Yin et al. (2012) | 253 | BMIz | Difference between control and center-based intervention only: B = −0.04; P > 0.1; difference between control and center-based + home intervention: −0.09; P < 0.09 | No effect | Unclear | Sampling bias not assessed; unclear if control were exposed to intervention |
|  |  | Weight-for-age z-score | Difference between control and center-based intervention only: B = −0.01; P > 0.1; difference between control and center-based + home intervention: −0.06; P < 0.04 | Positive | Unclear |  |
| Zaragoza-Cortes et al. (2019) | 19 | Length-for-age z-score | No effect; P = 0.53 | No effect | Unclear | Methods poorly reported |
|  |  | Weight-for-length z-score | No effect; P = 0.91 | No effect | Unclear |  |
